# *Streptococcus agalactiae cadD* alleviates metal stress and promotes intracellular survival in macrophages and ascending infection during pregnancy

**DOI:** 10.1038/s41467-022-32916-7

**Published:** 2022-09-14

**Authors:** Michelle L. Korir, Ryan S. Doster, Jacky Lu, Miriam A. Guevara, Sabrina K. Spicer, Rebecca E. Moore, Jamisha D. Francis, Lisa M. Rogers, Kathryn P. Haley, Amondrea Blackman, Kristen N. Noble, Alison J. Eastman, Janice A. Williams, Steven M. Damo, Kelli L. Boyd, Steven D. Townsend, C. Henrique Serezani, David M. Aronoff, Shannon D. Manning, Jennifer A. Gaddy

**Affiliations:** 1 Michigan State University, Department of Microbiology and Molecular Genetics, East Lansing, MI USA; 2 Aurora University, Department of Biology, Aurora, IL USA; 3 Department of Medicine, Vanderbilt University Medical Center, Nashville, TN USA; 4 Department of Medicine, University of Louisville, Louisville, KY USA; 5 Department of Pathology, Microbiology and Immunology, Vanderbilt University Medical Center, Nashville, TN USA; 6 Department of Pathology, Stanford University, Palo Alto, CA USA; 7 Department of Chemistry, Vanderbilt University, Nashville, TN USA; 8 Department of Medicine, Indiana University School of Medicine, Indianapolis, IN USA; 9 Department of Biomedical Sciences, Grand Valley State University, Allendale, MI USA; 10 United States Army Medical Research Institute of Infectious Diseases, Fort Detrick, MD USA; 11 Department of Life and Physical Sciences, Fisk University, Nashville, TN USA; 12 Department of Biochemistry and Structural Biology, Vanderbilt University, Nashville, TN USA; 13 Department of Obstetrics and Gynecology, Vanderbilt University Medical Center, Nashville, TN USA; 14 Center for Medicine, Health, and Society, Vanderbilt University, Nashville, TN USA; 15 Department of Veterans Affairs, Tennessee Valley Healthcare Systems, Nashville, TN USA

**Keywords:** Cellular microbiology, Bacterial immune evasion, Pathogens, Infection

## Abstract

Perinatal infection with *Streptococcus agalactiae*, or Group B *Streptococcus* (GBS), is associated with preterm birth, neonatal sepsis, and stillbirth. Here, we study the interactions of GBS with macrophages, essential sentinel immune cells that defend the gravid reproductive tract. Transcriptional analyses of GBS-macrophage co-cultures reveal enhanced expression of a gene encoding a putative metal resistance determinant, *cadD*. Deletion of *cadD* reduces GBS survival in macrophages, metal efflux, and resistance to metal toxicity. In a mouse model of ascending infection during pregnancy, the *ΔcadD* strain displays attenuated bacterial burden, inflammation, and cytokine production in gestational tissues. Furthermore, depletion of host macrophages alters cytokine expression and decreases GBS invasion in a *cadD*-dependent fashion. Our results indicate that GBS *cadD* plays an important role in metal detoxification, which promotes immune evasion and bacterial proliferation in the pregnant host.

## Abstract (web summary)

Perinatal infection with Group B *Streptococcus* (GBS) is associated with preterm birth, neonatal sepsis, and stillbirth. Here, Korir et al. show that gene *cadD*, encoding a putative metal efflux transporter, is important for metal detoxification, immune evasion and bacterial proliferation in the pregnant host.

## Introduction

Preterm birth affects 15 million pregnancies worldwide each year and is a significant cause of maternal and neonatal morbidity and mortality^1^. The most commonly associated cause of preterm birth, particularly early in gestation, is chorioamnionitis, or inflammation and/or infection of the placenta and fetal membranes^2^. Most often chorioamnionitis originates in the vaginal canal, and bacterial pathogens ascend to infect gestational tissues^3^. During infection, pathogens are recognized by pathogen recognition receptors leading to a cascade of proinflammatory changes within these tissues, which results in the trafficking of leukocytes that release metalloproteinases culminating in fetal membrane rupture, contractions and ultimately, preterm birth^3^.

Though chorioamnionitis is often a polymicrobial infection, several bacterial species have been identified as contributing to pregnancy-related infection outcomes. *Streptococcus agalactiae*, or Group B *Streptococcus* (GBS) accounts for 8–11% of all cases^4,5^. GBS is a common component of the gastrointestinal microbiota, and vaginal carriage is thought to result from transfer from the rectum^6^. During pregnancy 15–30% of women are colonized with GBS, and GBS vaginal colonization is a major risk factor for preterm birth, neonatal sepsis, and stillbirth^7–9^. Recent guideline updates from the American College of Obstetricians and Gynecologists continue to recommend routine rectovaginal screening for GBS between 36- and 38-weeks gestation, and administration of intrapartum antibiotic prophylaxis for those patients testing positive^10^. While these interventions have reduced the incidence of early-onset neonatal sepsis, GBS remains a leading cause of perinatal infections^11^. To develop new strategies to prevent and combat perinatal infections, a better understanding of mechanisms by which pathogenic bacteria ascend from the vaginal tract and invade gestational tissues are needed.

Fetally-derived macrophages that reside in gestational tissues, called Hofbauer cells or placental macrophages, play important roles in placental development, tissue remodeling, and controlling inflammation to support fetal development^12–14^. These cells assist in immune surveillance and combat invading microbes, but mechanisms by which these cells participate in host defense of the gravid reproductive tract remain poorly understood. Excessive inflammation within the reproductive tract can precipitate adverse pregnancy outcomes such as premature rupture of membranes and preterm birth^3^. Recent work by our laboratories has demonstrated that macrophages are recruited to sites of intrauterine infection in a mouse model of ascending chorioamnionitis^15^. Furthermore, we demonstrated that these macrophages show a polarization to a more proinflammatory M1 phenotype via GBS activation of the NLRP3 inflammasome and the transcription factor nuclear factor kappa beta (NFκβ)^16^. In general, macrophages kill bacteria, including *Streptococcus* species, via multiple mechanisms including phagocytosis, generation of reactive oxygen species, release of antimicrobial proteins, and extracellular trap release^17^. A less well-known mechanism by which macrophages kill bacteria is by accumulating transitional metals such as copper and zinc within phagolysomes to concentrations that are toxic to microbes^17,18^. Recently, GBS zinc and copper resistance mechanisms including the *sczA* and *czcD* loci and the *cop* operon have been discovered, and these are reported to be important for virulence in the vertebrate host^19,20^.

Not surprisingly, in response to innate immune responses, GBS has evolved mechanisms to survive host bactericidal strategies. We previously reported that GBS strains harbor virulence mechanisms that allow for prolonged survival within macrophages^21,22^. It has been proposed that pathogens able to readily persist within phagocytes may be more apt to disseminate into other tissues^23^. This may be particularly important during pregnancy as it remains unclear how GBS, a non-motile bacterium, traffics from the vagina across the cervix and into (and through) the gestational membranes in the pathogenesis of chorioamnionitis. We sought to understand cellular factors that impact GBS-macrophage interactions and determine their broader relevance to perinatal infections. In this work, we report that in response to interactions with macrophages, GBS cells express *cadD*, which encodes a putative metal efflux transporter critical for its intracellular survival. This system also confers resistance to numerous metal divalent cations and facilitates ascension of the reproductive tract during pregnancy and vertical transmission to the fetus in vivo.

## Results

### Ascending GBS infection of the reproductive tract results in bacterial–macrophage interactions

**Fig. 1:**
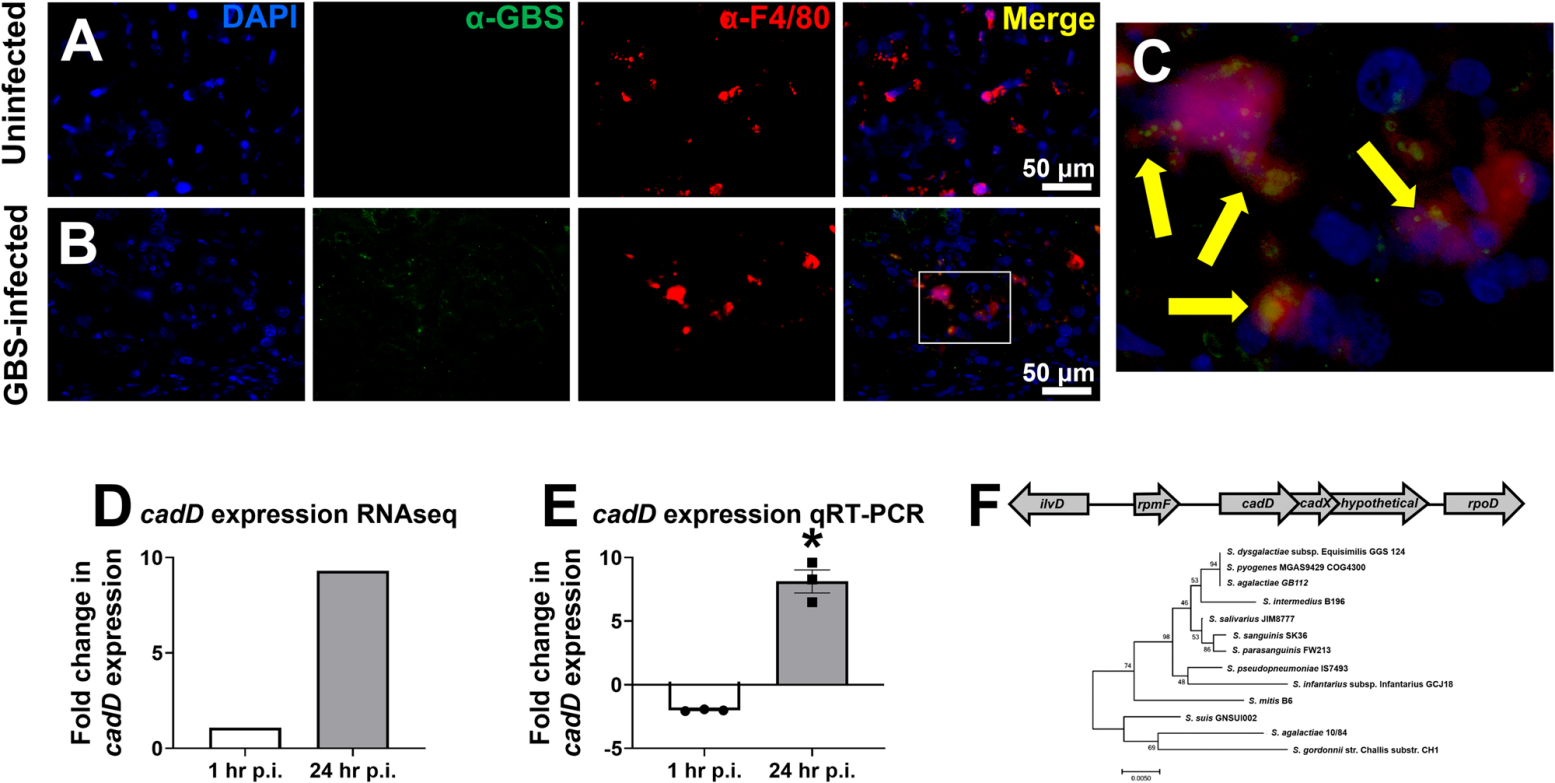
GBS localizes within tissue-resident macrophages and expresses *cadD* at high levels within macrophages. **A**–**C** Immunofluorescence microscopy examination of the placenta of either uninfected (**A**) or GBS-infected (**B**) mice reveals macrophages within the tissue as determined by staining with a monoclonal antibody to F4/80 (red). Tissues were also stained with a polyclonal antibody to GBS (green), and counter-stained with 4′,6-diamidino-2-phenylindole (DAPI; blue) to visualize cell nuclei. Merged images reveal co-localization of GBS and macrophages (yellow), magnification bar indicates 50 μm. **C** Enlargement of inset panel of the merged image in **B** indicates GBS cells associated with tissue macrophages within the placenta (yellow arrows). Micrographs are shown which are representative of images collected from 3 separate dams from each experimental group (uninfected or GBS-infected, *n* = 3). **D**, **E** GBS cultured within THP-1 macrophages in vitro were subjected to transcriptional analyses. **D** RNAseq analysis revealed that *cadD* was upregulated after 24 h of culture within macrophages, a result that was confirmed by quantitative real-time PCR (**E**), bars indicate mean and error bars indicate +/− SEM. *P = 0.0083, two-tailed, paired Student’s *t* test, *n* = 3 separate biological replicates. **F** Gene sequencing indicates that *cadD* is organized within a short operon and is highly conserved across numerous *Streptococcus spp*. including oral streptococci. The maximum likelihood phylogeny was constructed with 500 bootstrap replications; bootstrap support values are indicated at each node, scale bar indicates number of substitutions per site.

To identify critical cellular interactions that occur between host and pathogen during GBS infections of a pregnant host, we utilized a mouse model of ascending vaginal infection during pregnancy^24^. Pregnant dams were vaginally inoculated with GBS or with a sham treatment on embryonic day (E) 13.5 and reproductive tissues were collected after necropsy on E15.5. GBS strain GB112, a sequence type 17 strain previously examined in studies exploring interactions between GBS and decidual cells^25^ as well as macrophages^21^, was used. Placenta were analyzed via immunofluorescence microscopy (Fig. 1A–C) with GBS-specific antibody staining, macrophage-specific anti-F4/80 staining, and counterstained with a nuclear stain (4′,6-diamidino-2-phenylindole, DAPI)^15^. Tissue macrophages within GBS-infected cohorts were frequently found to harbor GBS within phagosome-like structures (Fig. 1B, C), and specific staining for GBS using a polyclonal rabbit antibody to GBS lysate in combination with a secondary antibody conjugated to Alexa 488 fluorophore (green), as well as macrophages using a monoclonal rat antibody to F4/80 in combination with a secondary antibody conjugated to Alexa 595 fluorophore (red) showed GBS colocalizing with macrophages (Fig. 1C), a result not observed in uninfected mice (Fig. 1A).

### The *cadD* locus is highly upregulated in response to bacterial interactions with macrophages

To identify GBS responses important to survive interactions with macrophages, GBS was co-cultured with THP-1 monocyte-derived macrophages for 24 h and bacterial RNA was collected and subjected to RNAseq analyses. Phorbol ester-differentiated THP-1 cells are an ex vivo cell line derived from a patient with acute monocytic leukemia, which are regarded as a valuable model for mimicking the function and regulation of macrophages^26^. Our prior published RNAseq analyses^27^ revealed that bacteria co-cultured with these macrophages resulted in a 9.8-fold upregulation in the GBS112 locus GB112_06484 (Fig. 1D). This locus is predicted to encode a putative cadmium transport protein sharing 60% sequence homology with the *Staphylococcus aureus cadD* locus, as determined by Basic Local Alignment Sequence Tool (BLASTx) analysis of the translated nucleotide sequence^28^. Hereafter, GB112_06484 will be referred to as “*cadD*”. Quantitative real-time (qRT)-PCR analyses confirmed the RNAseq results, demonstrating GB112 *cadD* expression is enhanced 8.1-fold in response to co-culture with macrophages compared to basal expression in planktonic GBS bacterial cultures (Fig. 1E).

### *cadD* resides in a putative operon and is highly conserved among *Streptococcus* spp

Whole genome sequence analysis revealed that the *cadD* locus in GB112 is upstream of two predicted bacterial transcriptional regulators, including one that shares 58% sequence homology with the *cadX* locus from *S. aureus* and a hypothetical protein, both of which have overlapping open reading frames that could indicate operonic arrangement. BLASTx analyses further revealed that the GB112 *cadD* locus shares 100% sequence identity with common laboratory strains including both A909 (accession # CP000114.1) and NEM316 (GCA_000196055.1). BLASTx also revealed that *cadD* is common among multiple streptococcal species and a maximum likelihood tree phylogenetic analysis indicates *cadD* is highly conserved within these species (Fig. 1F). GB112 *cadD* was phylogenetically compared to the 10/84 strain (GCA_000782855.1), *S. pyogenes* (CP000259.1), *S. gordonii* str. Challis substr. CH1 (CP000725.1), *S. mitis* B6 (FN568063.1), *S. suis* NSUI002 (CP011419.1), *S. infantarius* subsp. Infantarius CJ18 (CP003295.1), *S. pseudopneumoniae* IS7493 (CP002925.1), *S. parasanguinis* FW213 (CP003122.1), *S. sanguinis* SK36 (CP000387.1), *S. intermedius* B196 (CP003857.1), *S. salivarius* JIM8777 (FR873482.1), and *S. dysgalactiae* subsp. equisimilis (AP010935.1). Among the 12 genomes examined, the GB112 *cadD* was most closely related to sequences from *S. pyogenes* and *S. dysgalactiae* subsp. equisimilis, grouping together with 94% bootstrap support. GBS *cadD* (GB112_06484) shares 96% identity at the nucleotide level with the putative *cadD* loci in *S. pyogenes* MGAS9429 and *S. dysgalactiae*. BLASTx analyses indicate that the BLASTx analyses of non-streptococcal organisms reveal GB112 *cadD* locus also shares 99.5% sequence identity with a putative locus in *Mycobacterium tuberculosis* (WP_002911684.1) which is predicted to encode a CadD family cadmium resistance transporter, as well as 96% and 95% sequence identity with putative *cadD* loci in *Enterococcus cecorum* strain CE3 (CP010063.1) and *Neisseria meningitidis* NZ05/33 (CP002424.1), respectively. Taken together, these data indicate that putative *cadD* loci are highly prevalent among bacteria associated with vertebrate hosts with high levels of sequence similarities.

### *cadD* promotes GBS survival in macrophages

**Fig. 2:**
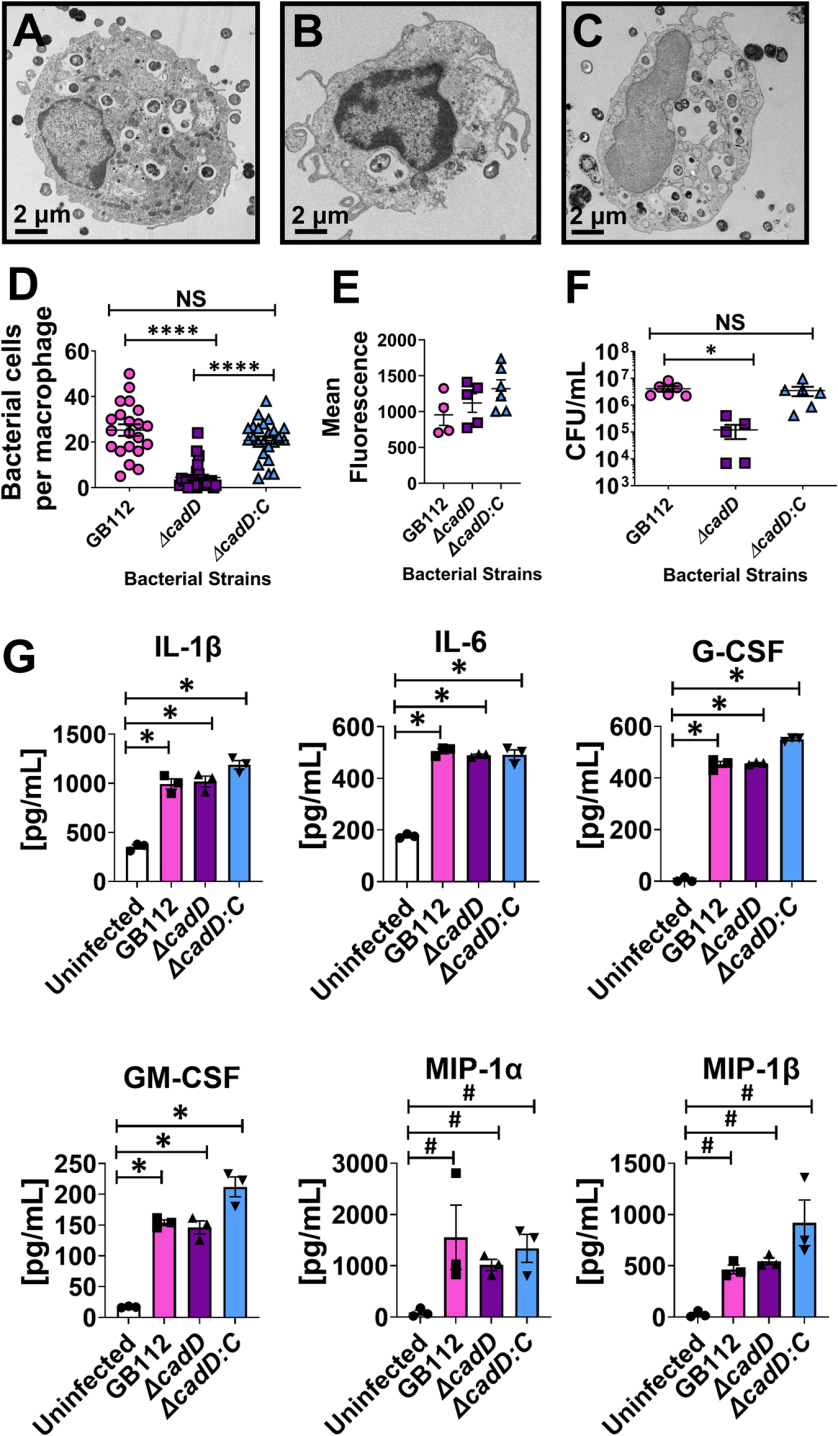
The *cadD* locus is required for GBS survival within human placental macrophages. **A**–**D** Transmission electron microscopy analyses of human placental macrophages in co-culture with WT GB112 (**A**), *ΔcadD* isogenic mutant (**B**), or the *ΔcadD:C* complemented isogenic mutant (**C**), quantification of GBS cells in macrophages (macrophages from 3 independent biological replicates were analyzed) by TEM analyses (**D**) indicates *cadD* is required for intracellular persistence and replication (****P < 0.0001, one-way ANOVA with Tukey’s post hoc multiple corrections test). Magnification bar indicates 2 μm. **E** Analysis of phagocytosis of GBS by primary human placental macrophages. GBS cells were labeled with the fluorophore FITC and co-cultured with placental macrophages for 3–4 h to allow phagocytosis to occur. Extracellular bacterial fluorescence was quenched with trypan blue and fluorescence was measured as a proxy for intracellular bacteria (mean fluorescence indicates fluorescence-background fluorescence of untreated placental macrophages). Gentamicin protection assay evaluation of intracellular bacterial viability (**F**). **D**–**F** Lines indicate mean +/− standard error mean. Co-cultures of GBS and placental macrophages were performed with WT GB112 (pink circles or pink bars), *∆cadD* isogenic mutant (purple squares or purple bars), and *∆cadD:C* complemented mutant (blue triangles or blue bars); uninfected controls are indicated as white bars (*P = 0.0183, one-way ANOVA with Tukey’s post hoc multiple corrections test). **G** Analysis of human placental macrophage secretion of cytokines (IL-1β, IL-6, G-CSF, GM-CSF, MIP-1α, and MIP-1β) in response to GBS infection (*P < 0.0001, one-way ANOVA with Dunnet’s post hoc multiple corrections test. ^#^*P* < 0.05 one-tailed, Student’s *t* test). All experiments were derived from three independent biological replicates (individual data points indicate macrophages derived different patient samples on different days). Bars indicate mean and error bars indicate +/− standard error mean.

Because *cadD* was highly upregulated in GBS co-cultured with macrophages, we sought to determine if it was necessary for survival within macrophages. An in-frame deletion of *cadD* was constructed to generate an isogenic GB112 *cadD* mutant (*ΔcadD*). Loss of *cadD* was complemented in trans using the pLZ12 shuttle vector^29^ to generate *ΔcadD:C*. Bacterial strains were co-cultured with placental macrophages and examined by transmission electron microscopy and quantitative techniques to evaluate GBS intracellular survival within macrophages (Fig. 2A–D). Although no difference was detected in placental macrophage phagocytosis of the three bacterial strains (Fig. 2E), bacterial survival within placental macrophages was attenuated in the *ΔcadD*-infected samples compared to cells infected with wild-type (WT) GB112 or complemented derivatives. Quantitation of bacterial cells per placental macrophage (20–35 macrophages per condition were analyzed) revealed WT-infected samples had an average of 25 bacterial cells per macrophage, whereas *ΔcadD*-infected samples had an average of 4 bacterial cells per macrophage (a significant decrease *P* < 0.0001, one-way ANOVA with Tukey’s multiple corrections test). This result was reversed through genetic complementation, which yielded an average of 21 bacterial cells per macrophage (Fig. 2D).

Bacterial intracellular survival assays were also performed (Fig. 2F)^30^. The *ΔcadD* isogenic mutant exhibited a 5.5-fold decrease in bacterial survival within placental macrophages (averaging 5.6 × 10^3^ bacteria per 10^5^ macrophages in 1 mL of media) compared to the parental WT strain (averaging 3.0 × 10^4^ bacteria per 10^5^ macrophages), a difference that was statistically significant (*P* = 0.0183, one-way ANOVA with Tukey’s multiple corrections test). Complementation of the *cadD* locus in trans restored bacterial survival to wild-type levels (1.3 ×1 0^5^ bacteria per 10^5^ macrophages). In addition, GBS co-culture with human placental macrophages resulted in significantly enhanced production of IL-1β, IL-6, G-CSF, and GM-CSF (*P < 0.0001, one-way ANOVA with Dunnet’s multiple corrections test) compared to uninfected cells regardless of *cadD* expression status, as determined by multiplex cytokine analysis (Fig. 2G). Similarly, expression of MIP-1α and MIP-1β was significantly induced by WT GB112 (*P* = 0.040 and *P* = 0.004, respectively, one-tailed Student’s *t* test), *∆cadD* mutant (*P* = 0.0008 and *P* = 0.00009, respectively, one-tailed Student’s *t* test), and the isogenic *∆cadD:C* complemented derivative (*P* = 0.005 and *P* = 0.008, respectively, one-tailed Student’s *t* test).

### *cadD* facilitates resistance to specific divalent metal cation challenge

**Fig. 3:**
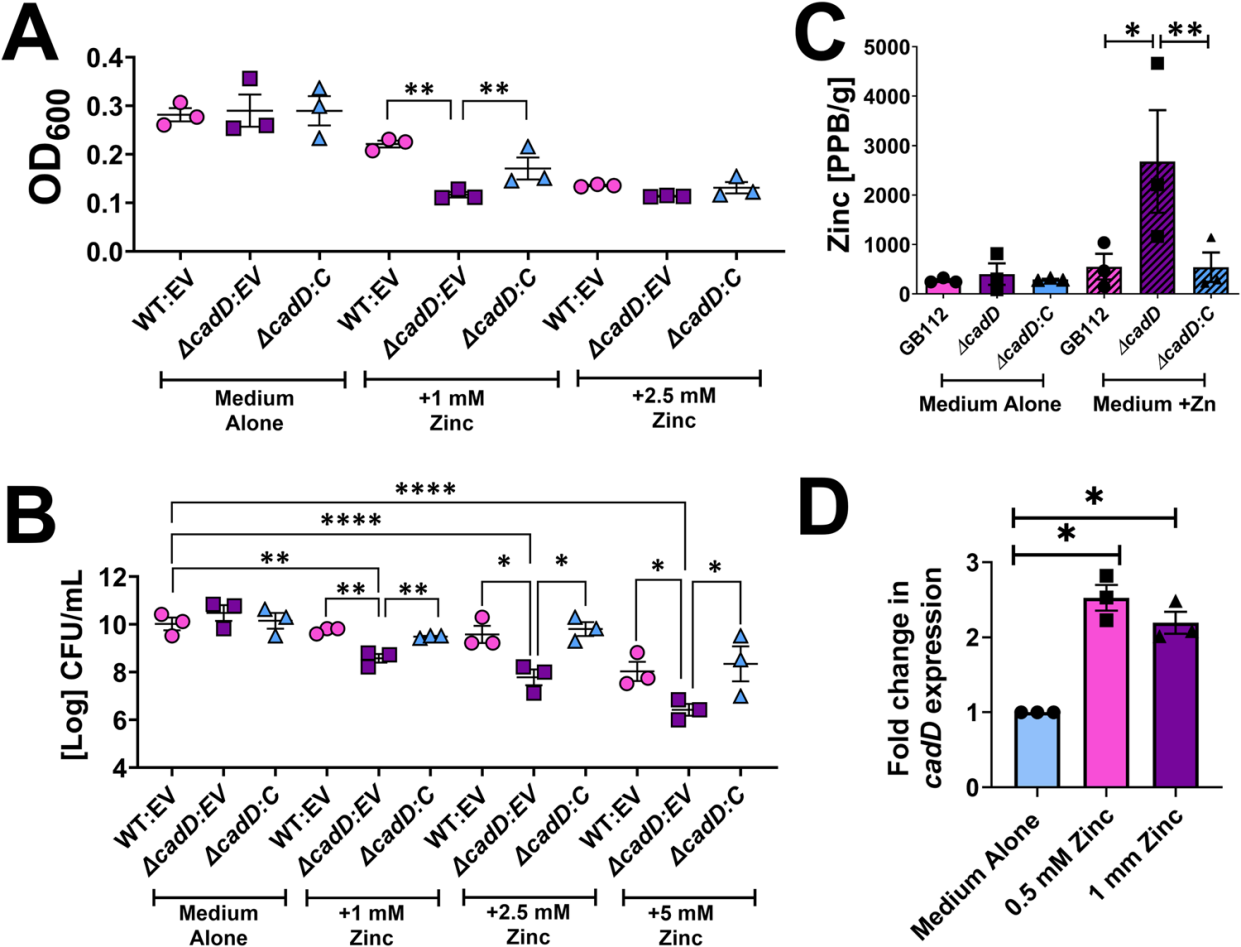
The *cadD* locus is implicated in zinc stress response and survival of zinc toxicity. **A** Analysis of wild-type parental GBS strain harboring the empty shuttle vector (WT:EV, pink circles), an isogenic *ΔcadD* mutant harboring the empty shuttle vector (*ΔcadD:EV*, purple squares), and complemented derivative (*ΔcadD:C*, blue triangles) final absorbance as determined by spectrophotometric determination of optical density at 600 nm (OD_600_) at 24 h post-inoculation in increasing concentrations of zinc (1 mM and 2.5 mM). **B** Analysis of bacterial viability (Log CFU/mL) in medium alone or supplemented with zinc (1 mM, 2.5 mM, or 5 mM). Bacterial cultures were grown overnight and subjected to serial dilution and plating for quantitative culture analyses. **C** Elemental analyses by ICP-MS of the WT (pink bars), isogenic *cadD* mutant (purple bars), or complemented derivative (blue bars) when grown in media supplemented with 100 µM zinc. **D** Real-time RT-PCR analyses of *cadD* expression in response to exposure to 0.5 mM zinc (pink bar) or 1 mM zinc (purple bar) compared to cells grown in medium alone (black bar). Bars indicate the mean of three independent biological replicates +/− SEM. *P < 0.05, **P < 0.01, ***P < 0.001, ****P < 0.0001, one-way ANOVA with Dunnet’s post hoc multiple corrections test compared to WT or medium alone controls. Individual data points indicate separate biological replicates.

Other bacterial pathogens, including *Mycobacterium tuberculosis*, utilize metal efflux determinants to survive within the phagosome^31^. Thus, we hypothesized that *cadD* facilitates GBS survival in macrophages by mediating resistance to divalent metal cation intoxication. To test this hypothesis, we performed growth curve analyses of WT GB112 harboring the empty shuttle vector pLZ12 (GB112), *ΔcadD* harboring the empty shuttle vector (*ΔcadD)*, and *ΔcadD:C* in increasing concentrations of metal cations including cadmium, calcium, cobalt, copper, iron, magnesium, manganese, nickel, or zinc (in the form of chloride salts from 0 to 7.5 mM). These concentrations span concentrations of metal ions (including copper and zinc) reportedly encountered by bacterial pathogens within the phagosome^32^. Isogenic derivatives harboring empty shuttle vectors were employed in this assay because chloramphenicol was required to maintain the complementation plasmid in these metal-stress assays, and GBS culture in the presence of this antibiotic impacted the growth of these strains in increasing concentrations of metal cations. Five metal cations exerted an enhanced toxicity phenotype in *ΔcadD*: zinc (Fig. 3A, B), cobalt, copper, nickel, and cadmium (Supplemental Fig. 1). As little as 10 µm cadmium chloride, 1 mM cobalt chloride significantly inhibited *ΔcadD* viability and cell density compared to the parental strain harboring the empty shuttle vector and the complemented strain. Similarly, exposure to 1 mM, or 2.5 mM copper chloride resulted in a 20% and 18% reduction in bacterial growth in *ΔcadD*, respectively, compared to the parental strain, a result that was reversed by complementation in trans. Exposure to 7.5 mM nickel chloride resulted in a 27% decrease in *ΔcadD* growth compared to the parental and complemented strains. Finally, as little as 1 mM of zinc chloride resulted in a 42% growth inhibition and 1.4-log viability decrease in the *ΔcadD* mutant compared to parental strain. Growth curve analyses (Supplemental Fig. 2) revealed that at 16 h post-inoculation, 5 mM zinc significantly inhibited both the parental strain harboring the empty shuttle vector, and the complemented ∆*cadD* isogenic mutant. At 24–36 h growth inhibition in these two strains was observed at 2.5 and 5 mM concentrations of zinc. Conversely, in the *∆cadD* isogenic derivative harboring the empty shuttle vector, growth inhibition was seen earlier, at 12 h, and at a lower concentration of zinc (0.75 mM, Supplemental Fig. 2). Specific metal hypersensitivity in *ΔcadD* was abolished by complementation of the *cadD* locus in trans. Together, these results indicate that the protein encoded by *cadD* provides promiscuous protection against nickel, cobalt, zinc, and copper intoxication.

### Inactivation of *cadD* results in the accumulation of divalent metal cations within GBS

Because *cadD* encodes a putative cadmium transport factor, we hypothesized this could serve as a divalent metal cation resistance factor such as an efflux determinant that operates to remove excess metal ions from the cell. To test this, we utilized inductively-coupled plasma mass spectrometry analysis (ICP-MS) to quantify the metal ions within GBS cells grown in medium alone or medium supplemented with exogenous metal sources (such as zinc, nickel, cobalt, or copper). ICP-MS demonstrated that in high zinc conditions, the *∆cadD* mutant had elevated zinc within its cells compared to the parental and complemented strains (Fig. 3C). Similarly, in conditions of high cobalt, copper, or nickel concentrations, *∆cadD* had elevated cobalt, copper, or nickel (respectively) within its cells compared to the parental and complemented strains (Supplemental Fig. 3).

### Environmental metal concentrations drive *cadD* transcriptional changes

To further test if *cadD* was an important factor in the GBS cell in response to metal challenge, we performed transcriptional analyses using RT-PCR on RNA extracted from GB112 cells exposed to concentrations of zinc that may be encountered in a macrophage cell (Fig. 3D). These results indicate that zinc challenge induces enhanced expression of the *cadD* locus compared to medium alone, implicating this gene as a zinc-responsive factor within the GBS cell.

### *cadD* is critical for GBS pathogenesis and cognate disease outcomes

**Fig. 4:**
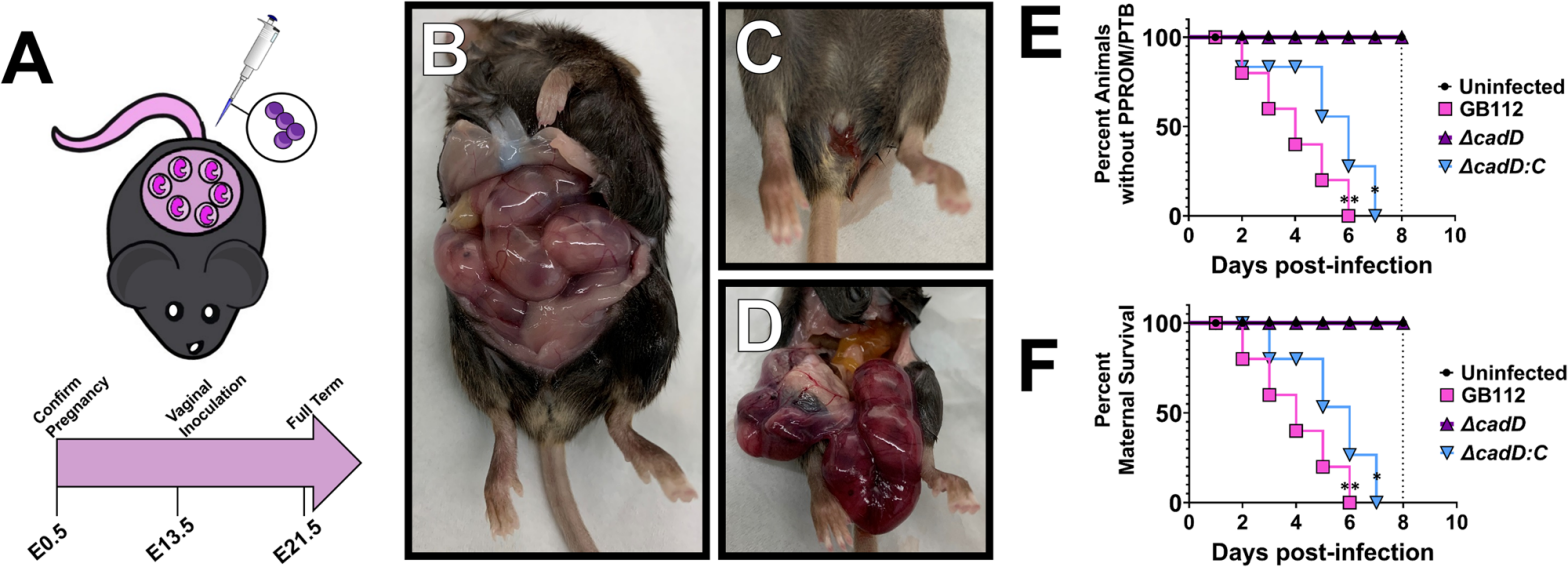
CadD contributes to GBS pathogenesis and cognate disease outcomes associated with infection. **A** Experimental design. Animals were harem mated and pregnancy was confirmed at embryonic day 0.5 by the presence of a mucus plug (E0.5). At embryonic day 13.5 (E13.5) animals were infected intravaginally with GBS. **B** Healthy pregnancy at 2 days post-infection vs. **C** premature rupture of membranes (PPROM) and **D** intrauterine hemorrhage at 2 days post-infection as a consequence of GBS infection. Analyses of percent animals **E** without PPROM or preterm birth (PTB) or **F** maternal survival. Dotted line indicates term for the average gestation in the C57BL6/J mouse model used in this study. *n* = 12 animals in the uninfected control group and 15 animals in the experimental groups. Infection with WT GB112 resulted in significant increases in PPROM and preterm birth (**E**) as well as maternal mortality (**F**) as determined by Mantel–Cox log-rank test (**P = 0.0024) and Gehan–Breslow–Wilcoxon test (**P = 0.0067). Animals infected with the *∆cadD* mutant experienced no observable PPROM, preterm birth or maternal mortality and were statistically indistinguishable from uninfected controls, a result that was reversed via genetic complementation (PPROM/PTB: Mantel–Cox log-rank test (*P = 0.0151) and Gehan–Breslow–Wilcoxon test *P = 0.0422 (**E**); maternal mortality: Mantel–Cox log-rank test (*P = 0.0147) and Gehan–Breslow–Wilcoxon test *P = 0.0339; compared to uninfected animals) (**F**).

Because the *∆cadD* mutant exhibited diminished ability to survive intracellularly within host innate immune cells, we sought to determine the role of *cadD* in disease outcome as a consequence of infection during pregnancy. To test this, we performed intravaginal infections^15,16,33^ with some modifications. We utilized an infectious dose of 5 × 10^3^ to 1 × 10^4^ CFU and allowed the infections to progress to assess endpoints including preterm premature rupture of membranes (PPROM), preterm birth, and maternal mortality (Fig. 4A). PPROM was observed as blood in or around the vagina, which could also be seen under necropsy within the uterus and around the fetuses (Fig. 4C, D), a result that is not commonly observed preterm in healthy control pregnancies (Fig. 4B). Preterm birth was assessed by the presence of neonates or pieces of neonates in the cages (as these are often consumed by dams). Infection with WT GB112 resulted in significant increases in PPROM and preterm birth (Fig. 4E) as well as maternal mortality (Fig. 4F) as determined by Mantel–Cox log-rank test (*P* = 0.0024) and Gehan–Breslow–Wilcoxon test (*P* = 0.0067). Interestingly, animals infected with the *∆cadD* mutant experienced no observable PPROM, preterm birth or maternal mortality and were statistically indistinguishable from uninfected controls, a result that was reversed via genetic complementation (PPROM/PTB: Mantel–Cox log-rank test (*P* = 0.0151) and Gehan–Breslow–Wilcoxon test *P* = 0.0422; maternal mortality: Mantel–Cox log-rank test (*P* = 0.0147) and Gehan–Breslow–Wilcoxon test *P* = 0.0339; compared to uninfected animals) (Fig. 4E, F).

### The *cadD* locus enhances ascending GBS infection in a pregnant mouse model of prenatal disease

**Fig. 5:**
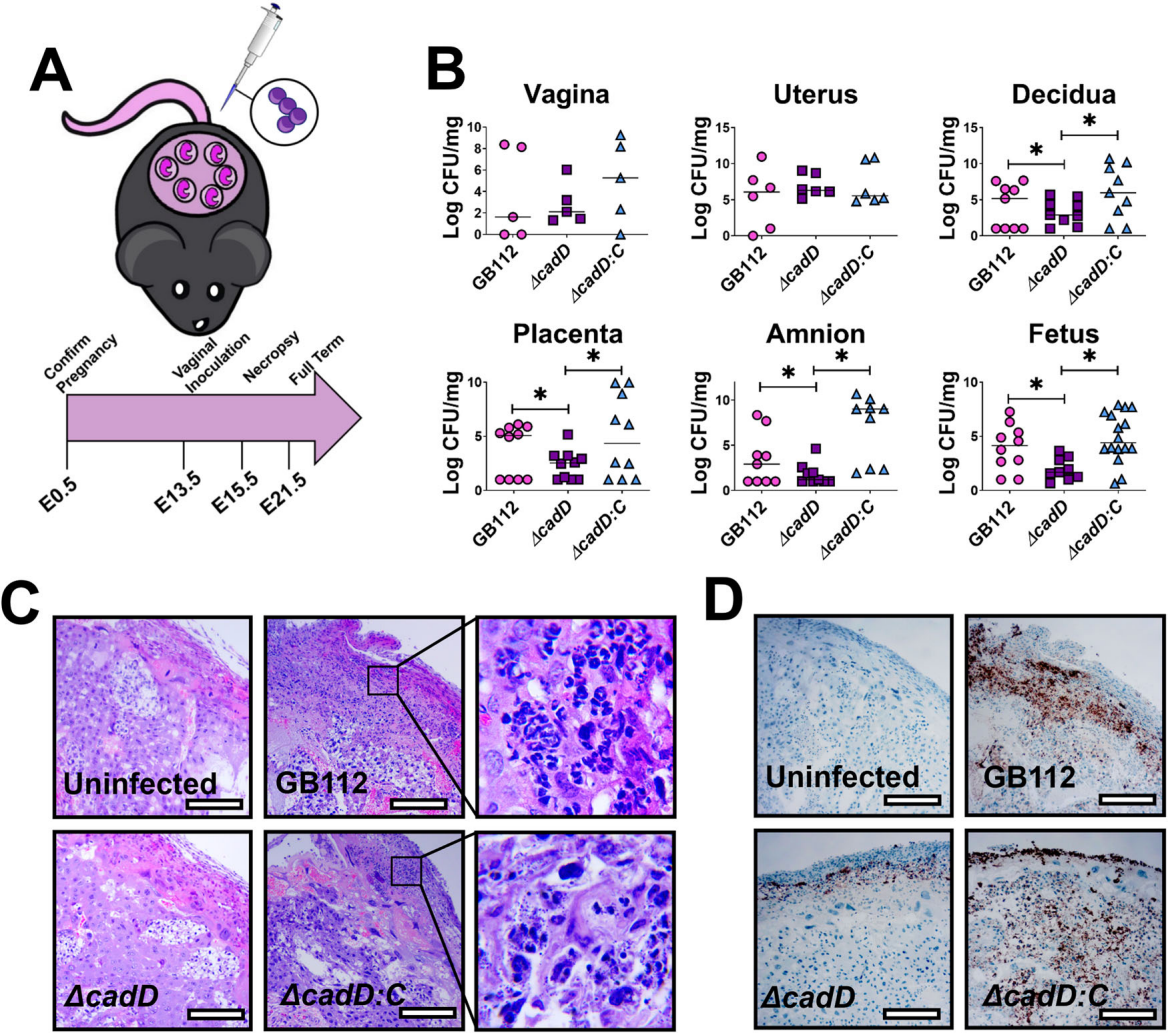
CadD plays a critical role in ascending GBS infection of a pregnant mouse. **A** Conceptual diagram of methods utilized in these studies. Pregnant mice were infected with GBS on embryonic day 13.5 and sacrificed 2 days post-infection. Reproductive tissues were collected for analyses. **B** Bacterial burden within reproductive tissues was evaluated by quantitative culture to determine Log CFU/mg (vaginal tissue was collected from 5 dams per experimental group, uterus tissues were collected from 6 separate dams, and all other tissues were collected from 6 separate fetal-placental units per experimental group). Significant changes in burden were observed between the WT GB112 and *∆cadD* mutant in the decidua (*P = 0.0257), placenta (*P = 0.0003), amnion (*P = 0.0312), and fetus (*P = 0.0266) by one-tailed Mann–Whitney U analysis. These results were reversed by genetic complementation in trans resulting in significant changes in burden observed between the *∆cadD* mutant and the complemented mutant in the decidua (*P = 0.0185), placenta (*P = 0.0285), amnion (*P = 0.0009), and fetus (*P = 0.0006) by one-tailed Mann–Whitney U analysis. **C** Histopathological examination of the hematoxylin and eosin-stained placental units at ×100 magnification (inset panel at ×400 magnification). **D** Placental units were analyzed by immunohistochemical techniques using a polyclonal antibody to stain specifically for GBS (indicated by the brown stain). Microscopic imaging of reproductive tissues from pregnant mice infected with WT GBS (GB112), isogenic *ΔcadD* mutant (*ΔcadD*), and the complemented isogenic *ΔcadD* mutant (*ΔcadD:C*). Micrographs are representative of analyses performed in a blinded fashion on tissues derived from 6 separate dams per experimental group. Magnification bars indicate 250 μm. All statistical analyses performed were one-tailed analyses.

To define the role of *cadD* in bacterial pathogenesis and host responses during GBS perinatal infections, we performed additional murine infections while utilizing an infectious dose of 5 × 10^2^ to 1 × 10^3^ CFU and sacrificing the mice 48 h post-infection (Fig. 5A). Reproductive tissues were collected for bacteriological and immunohistochemical (IHC) analyses. Amniotic fluid and maternal blood were also analyzed to evaluate bacterial dissemination, while gestational tissues were examined for disease by histopathological examination and developmental status (e.g., weight) (Supplemental Fig. 4). Although the *ΔcadD* mutant colonized the vagina and the uterus to similar quantitative levels as the parental strain (Fig. 5B), differences were observed for the placenta and decidua. Indeed, immunohistochemistry staining with an anti-GBS lysate antibody demonstrated that invasion into the placenta and decidua was attenuated in *ΔcadD* compared to the WT (Fig. 5D). Furthermore, *ΔcadD* exhibited decreased burden in the decidua (6.8-fold), placenta (10.2-fold), amnion (78.8-fold), and fetal tissues (3913-fold) compared to the parental strain. A 5-log decrease in bacterial dissemination to maternal blood was also observed in animals infected with *ΔcadD* compared to animals infected with the parental strain. The *ΔcadD* mutant exhibited diminished invasion into gestational tissues and a lower abundance of bacterial-associated staining; a result that was reversed by complementation in trans (Fig. 5B, D). In addition, GBS dissemination into the amniotic fluid and maternal blood was observed in pregnant animals infected with either WT or *∆cadD:C* complemented derivative but was not present in animals infected with the isogenic *∆cadD* mutant (Supplemental Fig. 4).

Because GBS causes placental inflammation in mouse models of infection during pregnancy^15,16,33^, tissues from uninfected and infected cohorts were analyzed by histopathological examination (Fig. 5C) and immunophenotyping (Fig. 5E and Supplemental Figs. 5–7). GB112-infected tissues had higher levels of inflammation characterized by infiltration of polymorphonuclear cells within the decidua and extending into the placenta, correlating with the spatial localization of GBS by both quantitative culture and IHC techniques (Fig. 5B–D). Tissues retrieved from animals infected with *ΔcadD* had sporadic infiltration of polymorphonuclear cells which were more abundant than control uninfected cohorts but were markedly decreased compared to GB112-infected cohort animals (Fig. 5C). Infection with the *cadD* complemented derivative also resulted in inflammation and polymorphonuclear cell infiltration within the placenta and decidua near the levels elicited by GB112.

### Proinflammatory cytokine secretion is enhanced in a *cadD*-dependent manner in a pregnant murine model of prenatal disease

Because the *ΔcadD* mutant showed decreased burden and neutrophilic infiltrates in the ascending model of vaginal infection during pregnancy, we hypothesized that this could be attributed to changes in inflammation and proinflammatory cytokine production as a consequence of bacterial infection. To test this, we utilized multiplex cytokine assays and enzyme-linked immunosorbent assays (ELISAs) to quantify the repertoire of cytokines and chemokines deployed by reproductive tissues in response to ascending GBS infections and compared them to uninfected control animal tissues. Our results indicate that infection with WT GBS results in significantly enhanced production of IL-1β, KC, and TNF-α in the uterus, decidua, placenta, amnion, and fetus (Supplemental Fig. 5) compared to uninfected controls (*P < 0.05, one-way ANOVA, ^#^*P* < 0.05, Student’s *t* test). Importantly, inactivation of the *cadD* gene resulted in a significant reduction in production of these cytokines and chemokines compared to WT-infected samples (*P < 0.05, one-way ANOVA, ^#^*P* < 0.05, Student’s *t* test). Similarly, G-CSF, MIP-1α, and MIP-1β were significantly upregulated in the uterus, decidua, placenta, and fetus (but not the amnion) of animals infected with WT GBS compared to uninfected animals (*P < 0.05, one-way ANOVA, ^#^*P* < 0.05, Student’s *t* test), and inactivation of the *cadD* gene resulted in a significant reduction in production of these cytokines and chemokines compared to WT-infected samples (*P < 0.05, one-way ANOVA, ^#^*P* < 0.05, Student’s *t* test). IL-6 and MIP-2 were upregulated in the uterus, decidua, placenta, and amnion (but not fetus) in response to WT GBS infection compared to uninfected controls, and inactivation of the *cadD* gene resulted in a significant reduction in production of these cytokines and chemokines compared to WT-infected samples (*P < 0.05, one-way ANOVA, ^#^*P* < 0.05, Student’s *t* test). Complementation with the wild-type *cadD* allele in trans restored cytokine and chemokine production similar to those observed in WT GBS-infected animals, or to levels that were significantly higher than those measured in the isogenic *∆cadD* mutant-infected samples (*P < 0.05, one-way ANOVA, ^#^*P* < 0.05, Student’s *t* test, NS = statistically indistinguishable from WT GBS).

Infection with WT GBS resulted in significantly enhanced production of GM-CSF, IL-7, IL-17, IP-10, LIF, LIX, MCP-1, and RANTES in the uterus (Supplemental Fig. 6) and eotaxin, GM-CSF, IL-1α, IL-15, IL-17, LIF, LIX, MCP-1, M-CSF, and MIG in the decidua (Supplemental Fig. 7) compared to uninfected controls (*P < 0.05, one-way ANOVA, ^#^*P* < 0.05, Student’s *t* test). Inactivation of the *cadD* gene, however, resulted in a significant reduction in production of these cytokines and chemokines compared to WT-infected samples (*P* < 0.05, one-way ANOVA, ^#^*P* < 0.05, Student’s *t* test).

### Macrophage depletion inhibits GBS invasion of reproductive tissues in a *cadD*-dependent manner

**Fig. 6:**
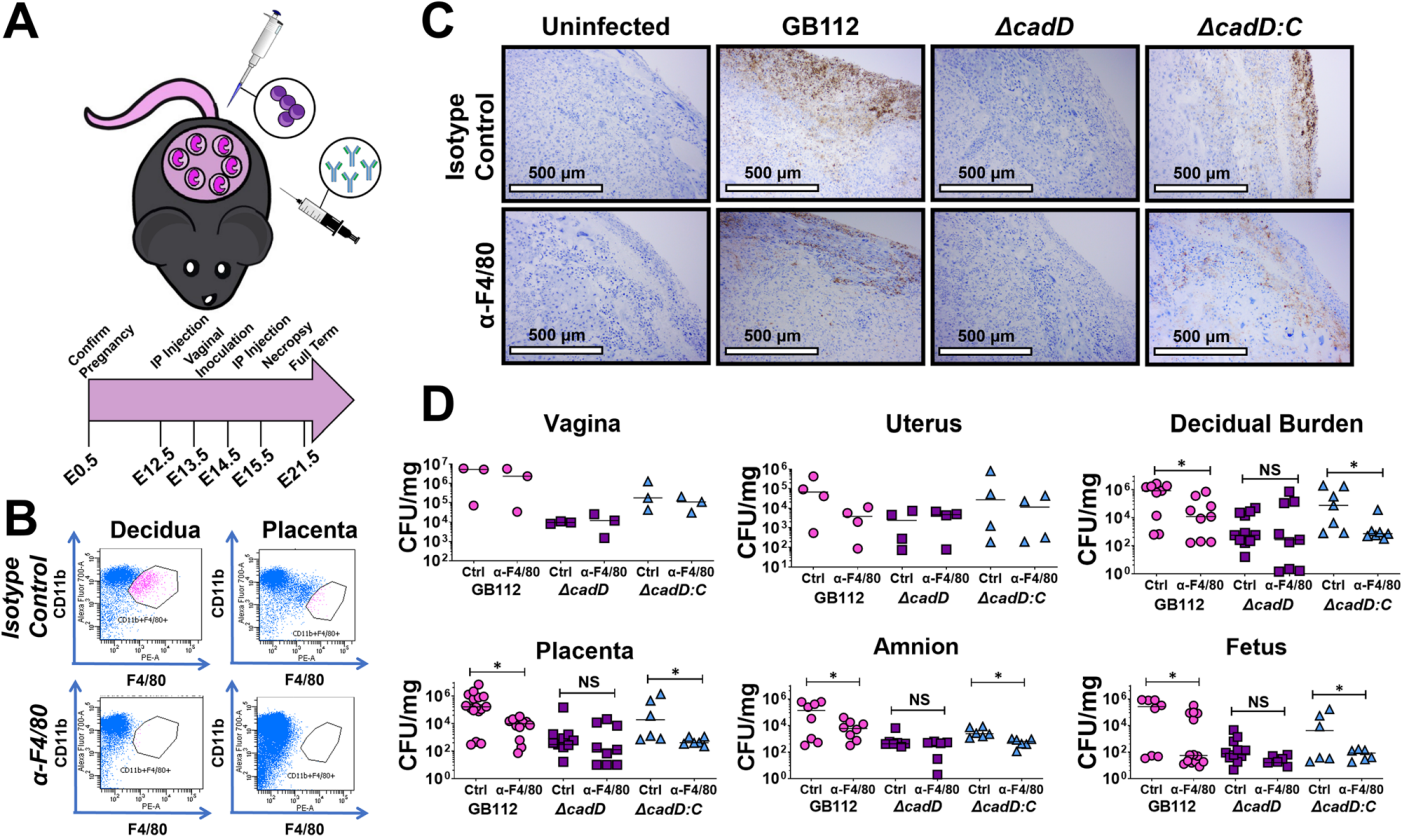
Depletion of host macrophages alters the outcome of GBS infection in a *cadD*-dependent manner. **A** Conceptual diagram of methods utilized in these studies. Pregnant mice were intraperitoneally injected on embryonic day 12.5 and 14.5 with either anti-F4/80 antibody to deplete host macrophages, or an isotype control antibody. Mice were infected with GBS on embryonic day 13.5 and sacrificed 2 days post-infection. Vaginal tissues were collected from three separate dams for each experimental condition for analyses. Uterine tissues were collected from four separate dams for each experimental condition for analyses. For decidual, placental, amnion, and fetal tissues, each individual data point indicates an independent fetal-placental unit. **B** Flow cytometry confirms that macrophages were depleted within the decidua and placenta at the maternal-fetal interface. **C** Placental units were analyzed by immunohistochemical techniques using a polyclonal antibody to stain specifically for GBS (indicated by the brown stain). Micrographs are representative of three biological replicates which were analyzed in a blinded fashion. Microscopical imaging at ×100 magnification reveals bacterial invasion of the reproductive tract in pregnant animals infected with WT GBS (GB112), the *ΔcadD* mutant (*ΔcadD*), and the complemented isogenic *ΔcadD* mutant (*ΔcadD:C*). Micrographs were analyzed in a blinded fashion and are representative of three biological replicates. **D** Bacterial burden within reproductive tissues was evaluated by quantitative culture (Log CFU/mg) in response to macrophage depletion via anti-F4/80 injections (+) compared to isotype control treatments (−) lacking macrophage depletion. Significant changes in burden were observed in WT GB112-infected mice between the isotype control and the anti-F4/80 treatment groups in the decidua (*P = 0.020), placenta (*P = 0.0008), amnion (*P = 0.0312), and fetus (*P = 0.0266) by one-tailed Mann–Whitney U analysis. These results were also observed in the cohort of animals infected with the complemented mutant in the decidua (*P = 0.0385), placenta (*P = 0.0130), amnion (*P = 0.0011), and fetus (*P = 0.0242) by one-tailed Mann–Whitney U test, but not in the animals infected with the isogenic *∆cadD* mutant (NS = not significant).

Because *cadD* was found to be important for GBS survival within macrophages, we hypothesized that depletion of host macrophages might potentially alter GBS-associated pathogenesis and cognate immune responses. To interrogate the role of macrophages in ascending GBS vaginal infection during pregnancy, we depleted macrophages by intraperitoneal injection with antibodies to F4/80 (anti-F4/80) on embryonic day 12.5 (E12.5) and 14.5 (E14.5); isotype control antibody treatments were used as a negative control (Fig. 6A). GBS infection was performed on E13.5 and necropsy was performed on E15.5 as in Fig. 5. Flow cytometry analyses confirmed that macrophages were depleted within the decidua and the placenta (Supplemental Fig. 8 and Fig. 6B). Analysis of bacterial burden by IHC (Fig. 6C) and quantitative culture (Fig. 6D) indicate that macrophage depletion significantly inhibited GBS invasion of the decidua, placenta, amnion, and fetus in both WT- and *ΔcadD:C*-infected cohorts; results that were not seen in the *ΔcadD* mutant-infected animals. This finding indicates that macrophages facilitate GBS penetration and proliferation in reproductive tissues and that this process requires expression of *cadD*.

### Macrophage depletion enhances proinflammatory cytokine production during GBS infection of a pregnant host in a *cadD*-dependant manner

Since macrophage depletion resulted in a lower bacterial burden in several tissue compartments of the gravid reproductive tract, we hypothesized that this could potentially influence immune responses, particularly proinflammatory cytokine production. Analysis of cytokines and chemokines within tissue compartments of the gravid reproductive tract revealed that macrophage depletion blunted MIP-1α, MIP-1β, and TNF-α in WT- or complemented *∆cadD:C* isogenic derivative-infected decidual tissues, but not in *∆cadD* mutant-infected tissues (Supplemental Fig. 9). In placental and decidual tissues, MIP-2 was blunted in WT-infected animals, a result that was not seen in animals infected with *∆cadD* mutant (Supplemental Fig. 9). Intriguingly, in the fetal compartment, IL-1β, KC, MIP-2, and TNF-α were enhanced in GBS-infected animals subjected to macrophage depletion treatments (Supplemental Fig. 9). Macrophage depletion was also associated with a significant enhancement of fetal resorption (Supplemental Fig. 10).

## Discussion

GBS has been a known perinatal pathogen since the 1970’s but there are still major gaps in our understanding of the pathophysiology of the infections and cognate disease outcomes caused by this organism. Based on the observation that GBS interacts with gestational tissue macrophages and can be found in phagosome-like structures in a gravid mouse model of ascending vaginal infection, we sought to understand factors that shape GBS-macrophage interactions and contribute to disease outcomes during pregnancy. We identified the expression of a putative metal efflux transport, *cadD*, to be upregulated when GBS was cultured with macrophages. Deletion of this locus resulted in impaired survival within macrophages and increased susceptibility to metals, which are known to be concentrated within the phagolysosome^30^. In our mouse chorioamnionitis model, mutants lacking *cadD* showed impaired invasion into gestational tissues and infection resulted in less inflammatory responses and disease compared to the parental strain or complemented mutant, demonstrating the importance of *cadD* for pathogenesis.

Macrophages represent the second most common leukocyte within fetal membranes tissues and are the major resident phagocyte^34^. These cells are thought to perform many roles including regulating tissue remodeling during development and modulating maternal-fetal tolerance^34^. Less is understood of the roles that macrophages may play during infection, as these cells are typically thought to be polarized to an anti-inflammatory M2 polarization in both human and mouse gestational tissues^14–16^. Some recent studies have noted that in response to ascending GBS infection, intrauterine macrophages change their polarization towards an M1 phenotype^16^.

Macrophages have also been implicated as a replicative niche for a variety of bacteria including *Pseudomonas aeruginosa*^35^, *Yersinia pestis*^36^, *Brucella neotomae*^37^, *Escherichia coli*^38^, *Neisseria gonorrhoeae*^39^, and *Legionella pneumophila*^40^. Recent work has demonstrated that *S. pneumoniae* can survive and replicate within splenic macrophages, which serve as a reservoir for septicemia^41^, and that Group A *Streptococcus* can survive and replicate within human macrophages^42^. These results mirror what we observed with GBS in placental macrophages. Furthermore, macrophages have been identified as a potential Trojan horse that aid in dissemination of a variety of pathogens including *Staphylococcus aureus*^43^, *Mycobacterium tuberculosis*^44^, *Chlamydia trachomatis*^45^, *Toxoplasma gondii*^46^, *Cryptococcus neoformans*^47^, and *Candida albicans*^48^. Similarly, depletion of macrophages has been shown to hamper *Chlamydia* dissemination in the reproductive tract^49^, underscoring the critical role that macrophages could play in the dissemination of pathogens in the human reproductive tract. Our results indicate that GBS can survive and proliferate within placental macrophages and peripheral blood macrophages, and that depletion of host macrophages inhibits dissemination in the reproductive tract, supporting the hypothesis that macrophages serve as a Trojan horse for GBS infections of the reproductive tract.

Bacterial survival within the macrophage requires overcoming a variety of antimicrobial stimuli^50^. One component of the macrophage antimicrobial armature against intracellular bacteria is metal intoxication^48^. When macrophages engulf bacteria by phagocytosis, they recognize bacteria via their pathogen-associated molecular patterns (PAMPs) by surface exposed, vesicular, or cytoplasmic pattern recognition receptors (PRRs)^50^. Pathogen recognition leads to inflammatory responses including secretion of cytokines, chemokines, small lipid mediators (SLM) as well as antimicrobial peptides (AMPs)^50^. Cytokines like GM-CSF enhance expression of two zinc transport proteins, which leads to accumulation of zinc ions in the Golgi apparatus and triggers the formation of toxic radicals by NADPH-oxidase, thereby exerting antimicrobial activity^51^. Similarly, IFN-γ induces the expression of the copper permease Ctr1 in macrophages, which results in increased uptake of copper into macrophages and translocation of the P-type ATPase ATP7A to phagolysosomes. This potentiates copper influx into the phagolysosome and subsequent metal poisoning of intracellular bacteria^52^. To circumnavigate metal toxicity, intracellular pathogens such as *M. tuberculosis*, employ metal efflux and detoxification systems including P-type ATPases, oxidases, and metal sequestration strategies^53–58^. Our work emphasizes that GBS must elaborate similar metal efflux strategies by using *cadD* (and potentially other factors) to evade divalent metal cation toxicity encountered within host macrophages. *cadD* is also highly conserved across streptococci and orthologs have been detected in other gram-positive pathogens such as *S. aureus*, providing further support that *cadD* confers resistance to metal stress^28,58^. In addition, recent work has demonstrated that *cadD* was underrepresented in a metal starvation assay using the zinc-binding host innate immune protein calprotectin to screen for isogenic derivatives in GBS implicated in the survival of metal starvation. Alongside the *cadD* locus, other putative zinc efflux determinants such as *sczA* and *czcD* were discovered indicating these could also be important factors in mitigating zinc stress^59^. A recent study demonstrates that GBS controls Zn export through CzcD to manage Zn stress and utilizes a system of arginine deamination to survive metal intoxication. Both of these systems are crucial for survival of GBS in vitro during zinc stress and also enhance virulence during systemic infection in non-pregnant mice^19^. In addition, the *cop* operon, including *copA* and *copY*, in GBS has been shown to play a critical role in survival of copper stress^20^. However, isogenic *czcD*, *sczA*, *copA*, and *copY* mutants were not attenuated in their survival within macrophage cells^19,20^, indicating the *cadD* locus might play an important role in this niche alone or in cooperation with these specific transporters. Thus, it is likely that GBS has redundant and overlapping mechanisms to handle metal toxicity, and that since *cadD* provides promiscuous protection against several divalent metal cations, it could play a versatile role in vivo alone or in tandem with these additional systems. Alternatively, the macrophage survival assays performed with the aforementioned isogenic *czcD*, *sczA*, *copA*, and *copY* mutants were performed with both human and mouse macrophage cells^19,20^, but our survival assays utilized primary human placental macrophages which could trigger metal intoxication pathways differently than the cells used in previous studies.

Our results demonstrate that deletion of the *cadD* locus has a wide impact on GBS pathogenesis, including inhibiting invasion of the gravid reproductive tract in vivo and induction of host proinflammatory cytokine production, phenotypes which were reversed by genetic complementation assays. Metal resistance has been shown to be critical for colonization of a vertebrate host in a variety of bacterial pathogens. For example, the CznABC system confers resistance to zinc, cadmium, and nickel in *Helicobacter pylori*. Isogenic *cznA*, *cznB*, and *cznC* mutants lack the ability to colonize the stomach in a Mongolian gerbil model, demonstrating that the metal export functions of *H. pylori cznABC* are essential for gastric colonization^60^. Similarly, *Listeria monocytogenes* utilizes CadC as a metal efflux determinant to escape the host response and promote infection in a mouse model of disease^61^. *cadC* also participates in the regulation of virulence in *L. monocytogenes* which, in turn, alters the repertoire of proinflammatory cytokines (such as TNF-α and IL-6) produced by the host in response to infection. Interestingly, although the *H. pylori* CznABC, *L. monocytogenes* CadC, and GBS CadD systems are involved in cadmium resistance, it remains unclear if macrophages or other innate immune cells utilize cadmium as an antimicrobial strategy. However, cadmium is recognized as a common heavy metal intoxicant that can be ingested or inhaled by a vertebrate host and has been shown to accumulate in immune cells and alter the function of the immune system including cytokine secretion and production of reactive oxygen species^62^. Cadmium exposure is commonly encountered through cigarette smoke exposure, and the biological half-life in humans is 10–35 years^62^, underscoring the potential influence of this non-essential metal. These findings, taken together, highlight the extraordinary importance of metal ion homeostasis for the bacterial survival and pathogenesis in the vertebrate host.

Our work demonstrates that survival in macrophages is critical for the promotion of GBS invasion into the gravid reproductive tract and induction of host inflammation via proinflammatory cytokine production including enhanced expression of IL-1β, KC, TNF-α, in the uterus, decidua, placenta, amnion, and fetus. Similarly, G-CSF, MIP-1α, and MIP-1β were significantly upregulated in the uterus, decidua, placenta, and fetus (but not the amnion), while IL-6 and MIP-2 were upregulated in the uterus, decidua, placenta, and amnion. Interestingly, *∆cadD*-infected animals had significantly less bacterial burden and proinflammatory cytokine production, a result that was reversed by genetic complementation assays. The upregulation of these proinflammatory cytokines paralleled with enhanced PPROM, preterm birth, and maternal mortality. Notably, previous work has associated elevated IL-1β, IL-8 (the human homolog of KC), IL-6, TNF-α, G-CSF, MIP-1α, MIP-1β, and MIP-2 with PPROM preterm birth^63–66^. Recent work indicates that blocking these proinflammatory cytokines (such as G-CSF) could have chemotherapeutic potential to inhibit inflammation-associated preterm birth^67^. Additional studies, however, are needed to better understand these relationships.

Macrophage depletion reduced bacterial burden and blunted MIP-1α, MIP-1β, MIP-2, and TNF-α in a variety of reproductive tissues in a *cadD*-dependent manner, indicating survival within macrophages via *cadD*-dependent metal resistance is critical for these cognate bacterial burden and immune response phenotypes. However, although macrophage depletion inhibited bacterial invasion and inflammation in some compartments, it resulted in enhanced IL-1β and KC in fetuses and was associated with significant enhancement of fetal resorption, indicating macrophages could play a critical role in anti-inflammatory activities in the fetal compartment. This result is congruent with those observed by Meng and colleagues, who reported enhanced fetal loss in mice subjected to macrophage depletion experiments^68^. Thus, macrophages are increasingly recognized as important immunoregulatory cells in the gravid reproductive tract and recent evidence indicates they promote maternal-fetal tolerance^68^.

**Fig. 7:**
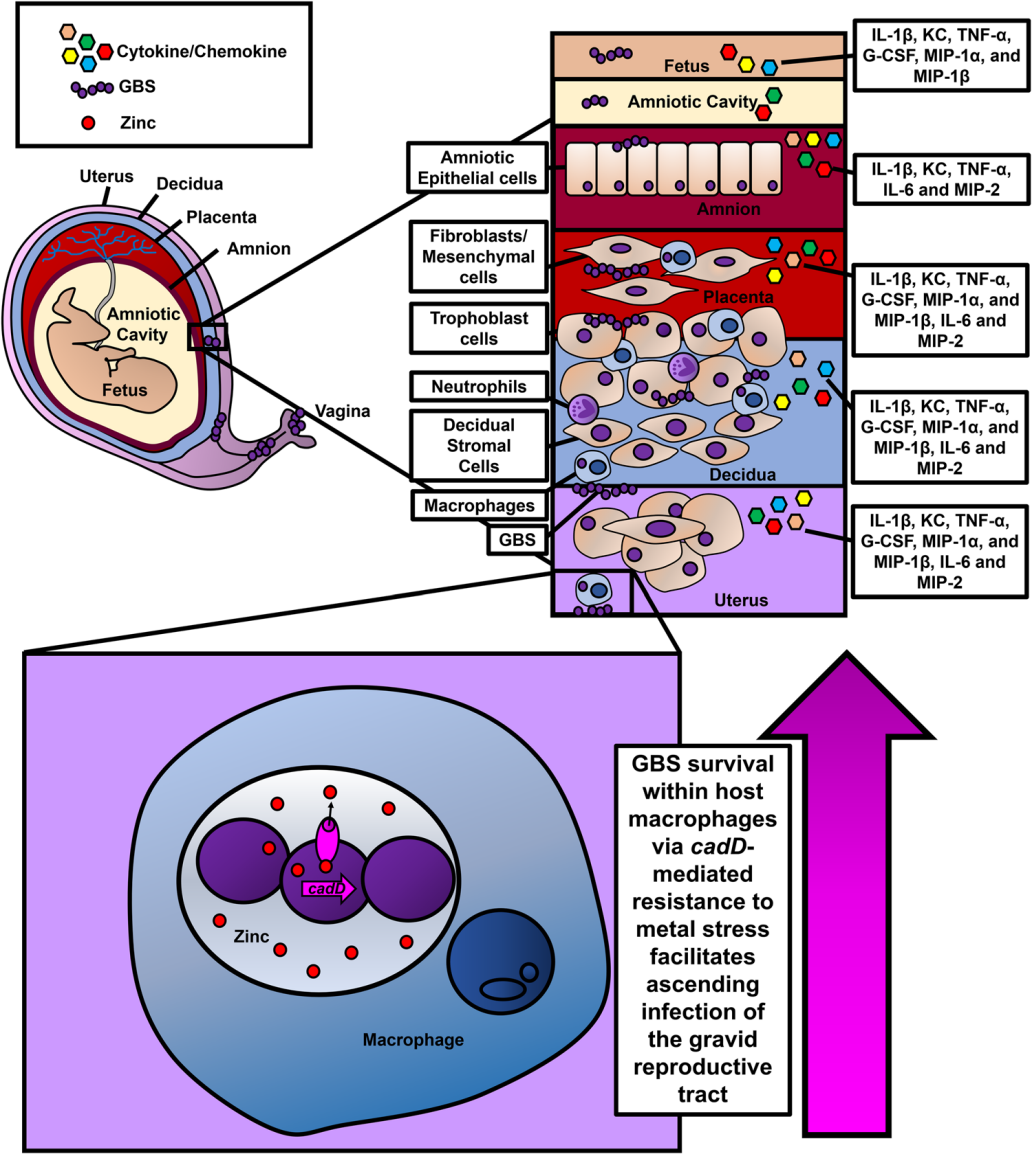
Conceptual model of the role of *cadD* in GBS pathogenesis. GBS infects the gravid reproductive tract and encounters sentinel innate immune cells such as macrophages within the tissues. Macrophages can perform phagocytosis to engulf GBS which deploys enhanced expression of *cadD* within the phagosome to overcome divalent cation stress, including zinc intoxication, to turn the macrophage intracellular space into a replicative niche. Replication and survival within macrophages aids in GBS penetrance and proliferation within the gravid reproductive tract. Concomitantly, ascending infection of the reproductive tract results in enhanced proinflammatory cytokine production in discrete tissue compartments.

Our study was hamstrung by limitations such as ex vivo culturing of human placental macrophages and immortalized THP-1 cells, which could vary in phenotype from in vivo tissue responses within a human host. Another limitation is the use of a mouse model which closely, but not perfectly, mimics human immune responses and displays structural and functional differences in reproductive tissues during pregnancy. Nonetheless, we have demonstrated that macrophages are critical innate immune cells that participate in immunoregulatory activities during pregnancy and are an Achilles heel that can be exploited by invading pathogens as a replicative niche. Upon encountering a macrophage, perinatal pathogens such as GBS can be engulfed by phagocytosis and circumnavigate the intracellular metal toxicity by elaborating the divalent metal cation efflux determinant CadD. CadD promotes GBS survival in macrophages, invasion of the gravid reproductive tract, and elicitation of proinflammatory cytokines which induce inflammation within reproductive tissues (Fig. 7). Understanding the role that virulence genes such as *cadD* play in GBS pathogenesis is critical for the development of novel therapeutic or prevention strategies aimed at decreasing the disease burden linked to GBS infections.

## Methods

### Ethics statement

This study was carried out in accordance with the recommendations of the Vanderbilt University Medical Center Institutional Review Board. This protocol was approved by the Vanderbilt University Medical Center Institutional Review Board (IRB #181998 and #00005756) and patients were enrolled in the study with written informed consent. All animal experiments were performed in accordance with the Animal Welfare Act, U.S. federal law, and NIH guidelines. All experiments were carried out under a protocol approved by Vanderbilt University Institutional Animal Care and Use Committee (IACUC: M/14/034 and M/17/012), a body that has been accredited by the Association of Assessment and Accreditation of Laboratory Animal Care Act (AAALAC).

### Cell lines, bacterial strains, and culture conditions

The GBS strain used in this study was GB00112 (GB112), which represents the wild-type or parental strain. This strain was isolated from a vaginal rectal screen of a patient who had recently given birth^69^ and was previously classified as capsule type III and sequence type (ST)-17 by multilocus sequence typing. It was previously shown to attach to and invade host cells and survive within host macrophages^21,22^. Isogenic mutants of this strain were generated using the primers and plasmids listed in Table 1, including a *cadD* deletion mutant (*∆cadD*) harboring the empty pLZ12 shuttle vector (*cadD:EV*), a complemented *∆cadD* mutant harboring a plasmid containing the *cadD* locus (*∆cadD:C*), and the parental strain harboring the empty vector alone (WT:EV), are listed in Table 2. Bacterial strains were grown on tryptic soy agar plates supplemented with 5% sheep blood (blood agar) plates or in Todd-Hewitt broth (THB) at 37 °C. Derivatives harboring the pLZ12 plasmid were grown in media supplemented with 3 µg/mL chloramphenicol. *E. coli* DH5α strains used for the mutation and complementation process were grown in LB broth or agar supplemented with either 150 µg/mL erythromycin or 20 µg/mL chloramphenicol when necessary.

**Table 1:** Oligonucleotide primers used in this study

Primer name	Sequence (5′ to 3′)
*cadD*-P1^a^	CCGC**GGATCC**CAGTACCTGCACGTCACACT
*cadD-*P2^b^	CCCATCCACTAAACTTAACAAAGCCCTGCCATCGAAATGA
*cadD-*P3^b^	TGTTTAAGTTTAGTGGATGGGTGCTATGGGCTGTGTTAGGC
*cadD*-P4^c^	GGG**GGTACC**CCTGCATTGCCTAGTTCGCT
*cadD*-P5	CAGTACCTGCACGTCACACT
*cadD*-P6	TACGTATAGCTCGAAGCGGT
*cadD-*pLZ12-F2^a^	CGC**GGATCC**AGGAGGACAGCTATGATTCAAAATGTTGTTAC
*cadD-*pLZ12-R2^d^	AAAA**CTGCAG**CTAGCCTAACACAGCCCATA
RT-*gyrA*-F	CGGGACACGTACAGGCTACT
RT-*gyrA*-R	CGATACGAGAAGCTCCCACA
RT-*cadD*-F	GCACAAGTCCCTTCTGTTGG
RT-*cadD*-R	GCCTAACACAGCCCATAGCA

^a^BamHI restriction site is shown in bold.

^b^Complementary sequences for P2 and P3 sets are underlined.

^c^KpnI restriction site is shown in bold.

^d^PstI restriction site is shown in bold.

**Table 2:** Strains used in this study

Strain ID	Strain name in this study	Description	Source or reference
GB00112	GB112 or WT	ST-17 GBS strain isolated from vaginal rectal swab of pregnant woman	22
GB02138	*ΔcadD*	GB00112 with *cadD* deletion	This study
GB02139	WT:EV	GB00112 with pLZ12 empty vector	22
GB02140	*ΔcadD:EV*	GB02138 with pLZ12 empty vector	This study
GB02141	*ΔcadD:C*	GB02138 complemented strain (*cadD_*pLZ12)	This study

Human leukemic monocyte THP-1 cells (ATCC TIB-202) were cultured in Roswell Park Memorial Institute (RPMI) 1640 growth medium (Gibco) supplemented with 2 mM L-glutamine, 10% fetal bovine sera (FBS; Gibco), and 2% penicillin/streptomycin (Gibco). Cultures were incubated at 37 °C in 5% CO_2_ supplemented room air. THP-1 cells were differentiated into macrophages by incubation with 100 nM phorbol 12-myristate 13-acetate (PMA; Sigma-Aldrich) and seeded with 1 × 10^6^ cells per well of a 24-well plate^22,70^.

### Generation of GBS competent cells and construction of isogenic bacterial mutant strains

GBS electrocompetent cells were generated as described with some modifications^71^. GBS was grown in THB with 0.5 M sucrose and a sublethal, but inhibitory concentration of glycine to early log phase (OD = 0.25) at 37 °C in shaking conditions. The culture was then pelleted at by centrifugation at 8000 × *g* at 4 °C, the supernatant removed, and the pellet washed with ice cold 0.625 M sucrose solution. The culture was pelleted again and resuspended in 0.625 M sucrose and stored at −80 ˚C. The *cadD* gene was deleted to generate the strain GB2138 (∆*cadD*) using the thermosensitive plasmid pG+host5^72,73^. The 5′ and 3′ flanking regions of *cadD* were amplified using the primer sets *cadD*-P1/*cadD*-P2 for 5′ and *cadD*P3/*cadD*-P4 for 3′ regions (Table 1). The two amplified flanking regions were combined in a crossover PCR, resulting in a single PCR product of the deletion sequence. The PCR product and the pG+host5 plasmid were digested with *Bam*HI and *Kpn*I then ligated to create pG+host51*cadD*. This plasmid was electroporated into GB112 competent cells and transformants were selected by growth on 2 µg/ml erythromycin at 28 ˚C. Cells in which the plasmid integrated into the chromosome via homologous recombination were selected for by growth on erythromycin at 42 ˚C. Colonies were sub-cultured into THB without antibiotic selection at 28 ˚C for several passages to allow for excision of the plasmid. The cultures were then plated and single colonies tested for erythromycin susceptibility and screened for gene deletion using PCR with primers *cadD*-P5 and *cadD*-P6. Deletion was confirmed by sequencing. Genetic complementation of the *cadD* deletion was done using the pLZ12 plasmid under control of the *rofA* constitutive promoter^74^. The coding sequence of *cadD* was amplified using the *cadD*-pLZ12 primer set (Table 1). The PCR product and the pLZ12-*rofA* plasmid were digested with *Bam*HI and *Pst*I and ligated to create *cadD*-pLZ12. The plasmid was electroporated into GBS competent cells and transformants were selected by growth on 3 µg/ml chloramphenicol.

### Mouse model of ascending vaginal GBS infection during pregnancy

GBS vaginal infection of pregnant mice were performed using C57BL6/J mice which were purchased from Jackson laboratories. Mice were housed using standard, solid-bottom, plastic cages in a room with 12-h light/dark cycle at ambient temperature of 21–22 °C 40–60% humidity. Mice were mated in harem breeding strategies (1 male to 3–4 females) overnight^22,31^. The following day, pregnancy was confirmed by the presence of a vaginal mucus plug establishing the embryonic date (E0.5). On embryonic day 13.5 (E13.5) pregnant dams were anesthetized via inhalation of isoflurane and vaginally infected with 5 × 10^2^–10^4^ colony forming units (CFU) in 0.05 mL of THB plus 10% gelatin. Uninfected controls were also maintained. For macrophage depletion experiments, animals were intraperitoneally injected with either anti-F4/80 antibody (16-4801-86; ThermoFisher) or the IgG2 isotype control (02-9602; ThermoFisher) on embryonic days 12.5 and 14.5. On embryonic day 15.5 (E15.5) animals were euthanized by carbon dioxide asphyxiation and necropsy was performed to harvest reproductive tissues including vagina, uterus, placenta, decidua, fetal membranes, and fetus.

### Histopathological analyses

Reproductive tissues were subjected to a primary fixation in 4% formalin (neutral buffered) overnight. The following day, tissues were embedded in paraffin and sectioned into 5-m-thick sections for staining and microscopical analyses. Sections were stained with hematoxylin and eosin for histopathological examination and imaged with an OMAX M83ES compound light microscope with ToupView software package.

### Immunohistochemical and immunofluorescence analyses

Tissues were fixed in 4% neutral buffered formaldehyde overnight before being embedded into paraffin blocks. Samples were cut into 5-μm sections, and multiple sections were placed on each slide for analysis. Samples were deparaffinized with xylene, and heat-induced antigen retrieval was performed on the Bond Max automated IHC stainer (Leica Biosystems) using Epitope Retrieval 2 solution for 5 to 20 min. Slides were incubated with a 1:100 dilution of rabbit polyclonal anti-GBS antibody (ab78846; Abcam) for 1 h. The Bond Polymer Refine detection system (Leica Biosystems) was used for visualization. Slides were counter-stained with eosin, dehydrated and cleared, and coverslips were added before light microscopy analysis was performed. For fluorescence microscopy, 1:250 dilution of rabbit polyclonal anti-GBS antibody (ab78846; Abcam) anti-F4/80 antibody (16-4801-86; ThermoFisher) was applied for 1 h, followed by 3 wash steps with PBS to remove unbound antibodies. Secondary staining was performed using a 1:100 dilution of either a goat-anti-rabbit antibody conjugated to an Alexa Fluor 488 fluorophore (ab150077; Abcam), and a goat-anti-rat antibody conjugated to an Alexa Fluor 594 fluorophore (ab150160; Abcam), which were applied for 1 h. The samples were washed three times with PBS, counterstained with 4′,6-diamidino-2-phenylindole (DAPI; ThermoFisher), mounted and viewed with an EVOS epifluorescence light microscope.

### Construction of the cadD-family phylogenetic tree

The CadD gene and amino acid sequences were obtained from streptococcal species with annotated *cadD* loci by using a BLASTx search of the nonredundant (nr) nucleotide database restricted to the *Streptococcus* genus in the National Center for Biotechnology Information (GenBank). The GB112 *cadD* nucleotide sequence was used for this tBLASTx search, and amino acid sequences associated with tBLASTx hits with an E value of ≤1e − 05 were retrieved. A single representative operational taxonomic unit from each retrieved species was used, amino acid sequences were aligned with MUSCLE software, and the *cadD* phylogenetic tree was reconstructed using maximum likelihood (ML) approach. The best-fit model of amino acid substitution used in the ML reconstruction was determined with ProtTest, version 2.4. The ML tree was reconstructed with PhyML, version 3.0, by maximizing the topology likelihood of 10 random starting trees from the best of nearest-neighbor interchange (NNI) and subtree pruning and regrafting (SPR) branch rearrangements. Amino acid equilibrium frequencies were set at empirical levels. Node statistical support was determined using a non-parametric bootstrap with 500 replicates^75^.

### RNA extraction, RNA sequencing, and transcriptional analyses

RNA isolation, cDNA synthesis, RNA sequencing (RNA-seq), and RT-PCR were performed to analyze GBS transcriptional responses^25,27^. Briefly, RNA samples were collected from bacterial culture in liquid medium by adding two volumes of RNAprotect Bacteria Reagent (Qiagen) or from bacteria inside macrophages by washing the cells twice with PBS then adding 1 mL RNAprotect Bacteria Reagent directly to the cells. RNA was extracted using the RNeasy minikit (Qiagen) using the “Enzymatic Lysis, Proteinase K Digestion, and Mechanical Disruption of Bacteria” protocol^22^. Residual genomic DNA was removed using the Turbo DNA-free kit (Ambion). To isolate RNA after infecting THP-1 cells, however, the MICROB*Enrich* Kit (Ambion) was used to deplete host cell RNA per the manufacturer’s instructions. The iScript Select cDNA synthesis kit was used to synthesize cDNA using random primers. As a control to test for DNA contamination, samples were processed without reverse transcriptase. qRT-PCR analysis was performed using the iQ SYBR Supermix (Bio-Rad) and gene-specific primers (Table 1). Fold change in gene expression was calculated using the 2^−∆∆CT^ method and relative transcript level was calculated using 2^−∆Ct^ method; *gyrA* was used as the internal control for both^76^.

For RNA sequencing, ribosomal RNA was removed from all samples using the Ribo-Zero rRNA Removal Kit for Gram-Positive Bacteria (Epicentre) according to the manufacturer’s directions. Libraries were prepared using the TruSeq Stranded mRNA Library Prep Kit (Illumina) at the Michigan State University Genomics Core; the protocol was modified to omit the oligo-dT bead step prior to fragmentation and first strand synthesis. The libraries were pooled and loaded into one lane of an Illumina HiSeq 2500 Rapid Run flow cell (v2) for paired-end (2 × 100 bp) sequencing using Rapid SBS reagents. Base calling was completed using the Illumina Real Time Analysis (RTA) v1.18.64 and the RTA output was demultiplexed and converted to FastQ files with Illumina Bcl2fastq, v1.8.4. The analysis was performed with the CLC Genomics Workbench (Qiagen) using the RNAseq analysis tool by mapping the reads to the published GB00112 genome (BioSample: SAMN01084069; Sample name: AKXO00000000). Differential expression analysis was performed using edgeR using the following comparisons: 1 h vs media alone, 24 h vs media alone, and 1 h vs 24 h. Genes with at least a 2-fold change in expression and *P* < 0.05 were considered to have significant differences in expression.

### Human placental macrophage isolation

De-identified placental tissue was collected from non-laboring women who delivered healthy, full-term infants by Caesarian section^77^. Briefly, a 30–60 g sample of tissue was excised from the placenta and washed three times in PBS, mechanically disrupted and enzymatically digested to a single cell suspension with DNase, collagenase, and hyaluronidase (Sigma-Aldrich). Cells were filtered and centrifuged, and CD14^+^ cells were isolated using the magnetic MACS Cell Separation system with CD14 microbeads (Miltenyi Biotec). Cells were incubated in RPMI 1640 medium (ThermoFisher) with 10% charcoal stripped fetal bovine serum (ThermoFisher) and 1% antibiotic/antimycotic solution (ThermoFisher) overnight at 37 °C in 5% carbon dioxide. The following day, PMs were suspended in RPMI 1640 medium without antibiotic/antimycotic and distributed into polystyrene plates. Cells were seeded at a density of 200,000 cells per well in a polystyrene, 24-well culture plate in RPMI with 1% antibiotic/antimycotic solution and 10% charcoal dextran FBS (RPMI+/+), and then incubated for 24 h in a humidified atmosphere at 37 °C and 5% CO_2_.

### Macrophage co-culture with GBS

Placental macrophages or PMA-differentiated THP-1 cells in RPMI 1640 medium without antibiotics were infected with GBS at a multiplicity of infection (MOI) of 10:1. Co-cultured cells were incubated at 37 °C in air supplemented with 5% carbon dioxide for 1 to 24 h. For the determination of GBS intracellular survival within macrophages, macrophages were inoculated at a MOI of 10:1 bacteria to host cells for 1 h. Co-cultures were washed with sterile media, resuspended in fresh medium containing 100 μg/mL of gentamicin (Sigma) to kill extracellular bacteria, and further incubated for 4 h at 37 °C. Gentamicin kills extracellular GBS but is limited in its ability to gain access to intracellular organisms. Subsequently, the samples were washed three times with sterile PBS, and cells were lysed by the addition of 1 mL of dH_2_O. Cellular cytoplasmic contents were serially diluted in PBS and plated to determine the number of viable intracellular bacteria. Samples containing only bacteria grown in RPMI, the same media used in the macrophage co-culture assays, were used to estimate the efficacy of antibiotic killing as a control experiment^78^.

### Transmission electron microscopy

Co-cultures of GBS and macrophages were subjected to primary fixation with 2.5% glutaraldehyde, 2.0% paraformaldehyde, in 0.05 M sodium cacodylate buffer at room temperature for 24 h. Subsequently, samples were washed three times with 0.05 M sodium cacodylate buffer and subjected to a secondary fixation step with 0.1% osmium tetroxide for 15 min. Samples were washed three times with 0.05 M sodium cacodylate buffer before being sequentially dehydrated with increasing concentrations of ethanol. After dehydration, samples were embedded in resin, polymerized, and sectioned into 70–90 nm sections via ultramicrotomy. Sections were lifted onto nickel or copper 100 mesh grids (Electron Microscopy Sciences) and secondarily stained with 1% phosphotungstic acid. Grids were imaged with a Philips/FEI T12 transmission electron microscope to visualize intracellular bacteria. Bacteria were enumerated by blinded analysis using ImageJ software package.

### GBS growth and viability analyses

For growth and viability analyses, bacteria were cultured overnight in THB (to an OD_600_ of ∼0.7), then sub-cultured by performing a 1:100 dilution (roughly 7 × 10^6^ CFU/mL) in fresh THB referred to as “medium alone” or medium supplemented with increasing concentrations (100, 250, 500, 750, 1000, 2500, 5000 µM) of zinc chloride, cobalt chloride, copper chloride, nickel chloride, iron chloride, magnesium chloride, calcium chloride or (1, 2.5, 5, 10, 25, 50 µM) of cadmium chloride. Bacterial growth was evaluated at 0, 4, 12, 16, 24, and 36 h post-inoculation by spectrophotometric reading of OD_600_ or bacterial viability was evaluated at 24 h by serial dilution and plating onto blood agar plates and quantifying viable colony forming units per mL of culture (CFU/mL) by spotting 3 µL of serial diluted (10^−1^ to 10^−8^) overnight cultures in fresh THB medium. Plates were incubated overnight at 37 ˚C and imaged and quantified the following day to determine bacterial survival in increasing concentrations of metal stress. In addition, bacterial growth on THB agar plates alone or supplemented with the aforementioned metal concentrations was also evaluated by serial diluting (10^−1^ to 10^−8^) overnight cultures in fresh THB medium then spotting 3 µL of culture onto THB agar alone or agar supplemented with cadmium, zinc, copper, cobalt, or nickel, incubated overnight and colonies were analyzed the following day.

### ICP-MS elemental analysis

To enumerate metal ions associated within GBS bacterial cells 5 mL of bacterial cultures of each strain (GB112, *∆cadD*, or *∆cadD:C*) were grown overnight in THB alone or supplemented with 100 µM or zinc, nickel, cobalt or copper (chloride salts). Cells were pelleted by centrifugation at 8000 × *g* for 15 min and washed three times with ethylenediaminetetraacetic acid (EDTA) buffer (10 mM, pH 7.4) to remove any extracellular metals present. Subsequently, each sample was centrifuged at 8000 × *g* for 15 min, supernatant was decanted, and the resulting pellet was weighed and digested in 0.9 mL 50% nitric acid overnight at 37–50 °C in sealed Teflon sample containers. The following day, samples were diluted in 9.1 mL of deionized water in metal-free tubes. Elemental quantification was performed using a Thermo-Element 2 HR-inductively coupled plasma mass spectrometry (ICP-MS) ESI autosampler instrument. The ICP-MS was performed with a PFA microflow nebulizer, double channel spray chamber, tandem magnetic sector, and electric sector followed by a second electron multiplier. Sample uptake was performed via self-aspiration with a 0.5 mm ID sample probe and capillary which facilitated detection of zinc, cobalt, copper, and nickel.

### Quantifying bacterial burden in host tissues

To determine bacterial burden in reproductive tissues quantitative culture methods were employed^33^. Briefly, reproductive tissues were weighed and placed in 1 mL of sterile THB. Tissues were homogenized using an electric tissue tearor (Cole-Parmer) and subjected to serial dilution and plating onto blood agar to enumerate bacteria (CFU/mg) in host tissue.

### Cytokine analyses

Mouse reproductive tissues, maternal sera, and amniotic fluid were analyzed by multiplex cytokine assays. Mouse tissues were placed in 1 mL of sterile PBS or THB + 10 mg/mL penicillin and homogenized and passed through a 0.22 µm filter. Samples were frozen at −80 ˚C or on dry ice until analyses were performed. Samples were analyzed by Eve Technologies via multiplex cytokine array (Eve Technologies, Alberta, Canada^79^. Validation of host targets for specific cytokines (IL-1β, IL-6, KC, and TNF-α) were performed by sandwich ELISA (AbCam)^80^.

### Flow cytometry

Four to seven placenta and decidua were collected from each animal (4–6 animals total) and pooled, weighed, minced with sterile scissors and digested in a solution containing 1 mg/mL collagenase, 1 mg/mL hyaluronidase, and 150 µg/mL DNAse I with agitation for 1 h at 37 °C^33,77^. A total of 40 mL of digestion solution was used per sample (each sample consisted of a single animal’s pooled placenta or decidua, totaling 4–7 per animal) and samples were washed using RPMI+/+ medium (containing 1% antibiotic and 10% fetal bovine serum), centrifuged at 1500 RPM at 4 °C for 10 min followed by 100 µm nylon mesh filtration to eliminate remaining particulates. Cells were then washed and the filtrate was resuspended in 25% Percoll in RPMI+/+, overlaid onto 50% Percoll, with 2 mL PBS layered above the 25% Percoll. Percoll gradient was centrifuged for 35 min at 1500 RPM and cells were washed as before, followed by 10 min at room temperature in RBC lysis buffer (eBiosciences). After two more washes with RPMI+/+, cells were enumerated and one million cells were aliquoted into flow cytometry tubes. Cells were surface stained for 20 min at 4 °C with a 1:100 dilution of anti-mouse CD45, anti-mouse CD11b, anti-mouse F4/80, and FcR blocking reagent. Isotype controls were included in separate tubes. After surface staining, all cells were washed with PBS and stained with a live/dead viability dye (Life Technologies) for 30 min at 4 °C. Cells were then washed with 4 mL PBS containing 1% BSA (FACS buffer), fixed for 15 min at 4 ˚C with 1% paraformaldehyde in PBS, and washed again with FACS buffer. Cells were resuspended in FACS buffer and immediately acquired on a BD LSR2 Flow Cytometer (BD Biosciences). Analyses were performed with BD FACS Diva software (BD Biosciences).

### Phagocytosis assay

Placental macrophages were cultured in fresh RPMI medium alone or with a 10:1 inocula of bacterial cells that were pre-treated with FITC stain^15^. Co-cultures were incubated for 3–4 h before being washed three times with sterile PBS. Extracellular bacterial fluorescence was quenched with trypan blue stain, and intracellular fluorescence was measured at an excitation wavelength of 495 nm and emission at 519 nm to ascertain the fluorescence intensity as a proxy for intracellular bacterial presence within placental macrophages^15^.

### Statistics

Statistical analysis of parametric data with more than two groups was performed using one-way ANOVA with either Tukey’s or Dunnet’s post hoc correction for multiple comparisons; all reported *P* values were adjusted to account for multiple comparisons. For parametric data with two groups, a Student’s *t* test or one-way ANOVA were used. *P* values of ≤0.05 were considered significant. Non-parametric data (such as CFU data) were analyzed by one-tailed Mann–Whitney U test. Growth curve analyses were performed using two-way ANOVA. All data analyzed in this work were derived from at least three biological replicates (representing different placental samples). Statistical analyses were performed using GraphPad Prism 9 (GraphPad Software Inc.).

### Reporting summary

Further information on research design is available in the Nature Research Reporting Summary linked to this article.

## Supplementary information

Supplementary Information

Reporting Summary

## Data Availability

RNA-Seq data were deposited in the Gene Expression Omnibus (GEO) under Accession numbers GSE184709. The authors declare that all other data supporting the findings of this study are available within the Source data file and Supplementary Information files, or from the corresponding authors upon request. Source data are provided with this paper.

## Source data

Source Data
