# Supplementary material for: Streptococcus agalactiae cadD alleviates metal stress and promotes intracellular survival in macrophages and ascending infection during pregnancy
Source: Nat Commun. 2022 Sep 14;13:5392. doi: 10.1038/s41467-022-32916-7 (PMC9474517; doi:10.1038/s41467-022-32916-7)
Supplement: Supplementary file 1 — Supplementary Information [file 41467_2022_32916_MOESM1_ESM.pdf]

Supplemental Figures and Legends

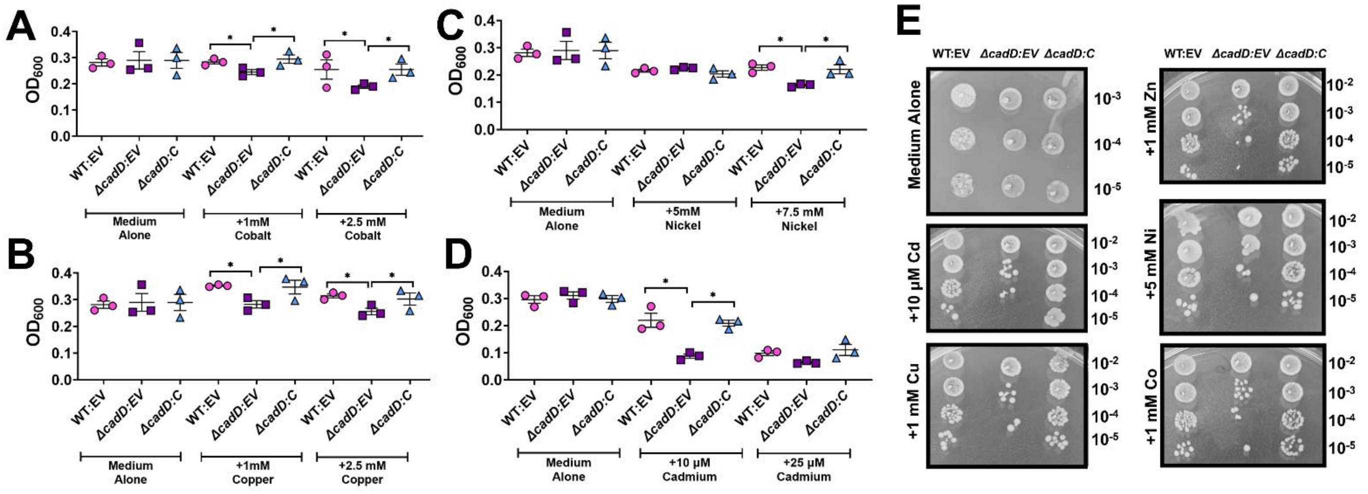

Supplemental Figure 1. The *cadD* locus is implicated in GBS cell density and viability in the presence of metal toxicity. Analysis of culture turbidity as determined by spectrophotometric determination of optical density at 600 nm (OD_{600}) at 24 hours post-inoculation in increasing concentrations of A) cobalt, B) copper, C) nickel, or D) cadmium. Results indicate the wild-type parental GBS strain harboring the empty shuttle vector (WT:EV, pink circles) grows well in high metal concentrations. However, an isogenic *ΔcadD* mutant harboring the shuttle vector (*ΔcadD:EV*, purple squares) is attenuated in growth within liquid culture at 1 mM and 2.5 mM of cobalt and copper, and 7.5 mM nickel, and 10 μM cadmium, a result that was reversed by genetic complementation assays (GB112 *ΔcadD:C,* blue triangles). Lines indicate mean, error bars indicate +/- SEM, individual data points indicate independent biological replicates. *P<0.05, paired, one-tailed Student's *t* test. E) Analysis of bacterial growth after serial dilution and spotting 3 microliters of culture onto semisolid medium (Todd-Hewitt Agar) alone (medium alone) or supplemented with zinc (Zn), copper (Cu), cobalt (Co), nickel (Ni) or cadmium (Cd). Images are representative of three independent biological replicates.

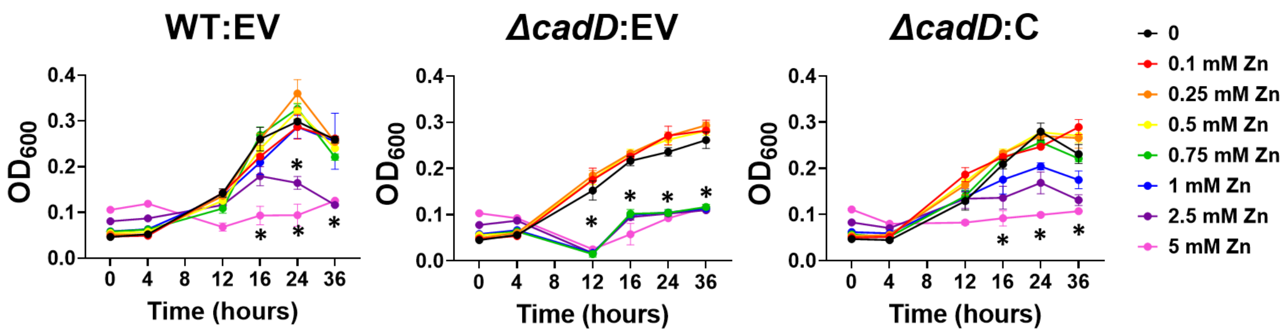

Supplemental Figure 2. The *cadD* locus is implicated in GBS growth under conditions of zinc toxicity. Analysis of culture turbidity as determined by spectrophotometric determination of optical density at 600 nm (OD_{600}) at 0, 4, 12, 16, 24, and 36 hours post-inoculation in increasing concentrations of zinc chloride. Wild-type parental GBS strain harboring the empty shuttle vector (WT:EV) exhibits significant growth inhibition at 16 hours at a concentration of 5 mM zinc, and at 24-36 hours at 2.5-5 mM zinc concentrations. However, an isogenic *ΔcadD* mutant harboring the shuttle vector (*ΔcadD:EV*) exhibits attenuated growth earlier (at 12 hours post-inoculation) and at lower concentrations of zinc (0.75 mM zinc chloride), a result that was reversed by genetic complementation assays (*ΔcadD:C*). Points indicate mean, error bars indicate +/- SEM, n=3 independent biological replicates. *P<0.05, two-way ANOVA comparison to same timepoint medium alone control (0 Zn).

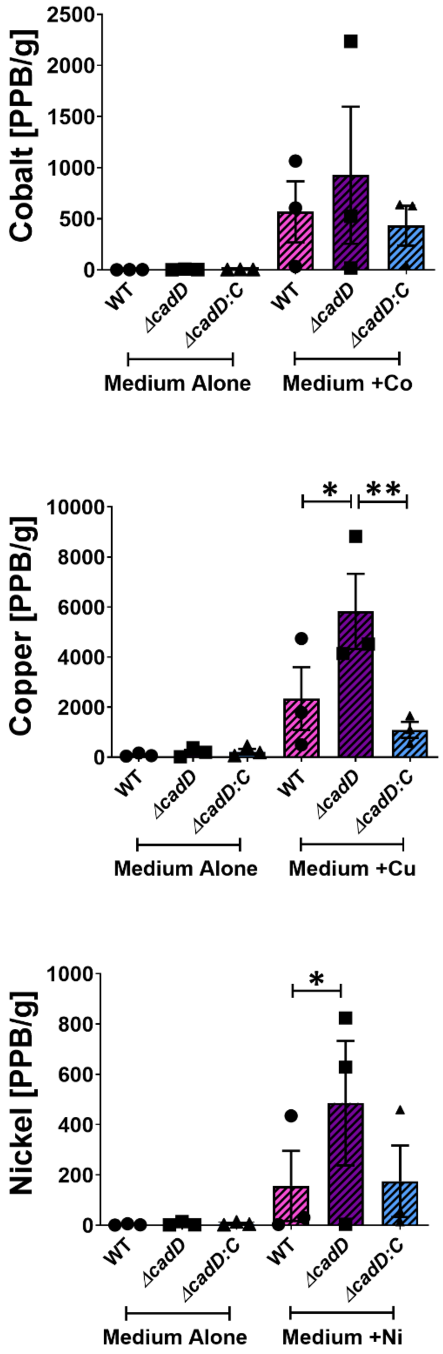

Supplemental Figure 3. Elemental analysis of cell-associated metal ions. Analysis of bacterial strains WT GB112 (pink bars), Δ*cadD* isogenic mutant (purple bars), and Δ*cadD:C* complemented mutant (blue bars) grown in medium alone (open bars) or medium supplemented with exogenous metal ions (hatched bars) were analyzed by ICP-MS to enumerate metal ions (cobalt= Co, nickel=Ni, or copper= Cu) within cells. Bars indicate mean +/- SEM. *P<0.05, **P<0.01, one-way ANOVA, with a Tukey's multiple corrections post hoc test, n=3 independent biological replicates.

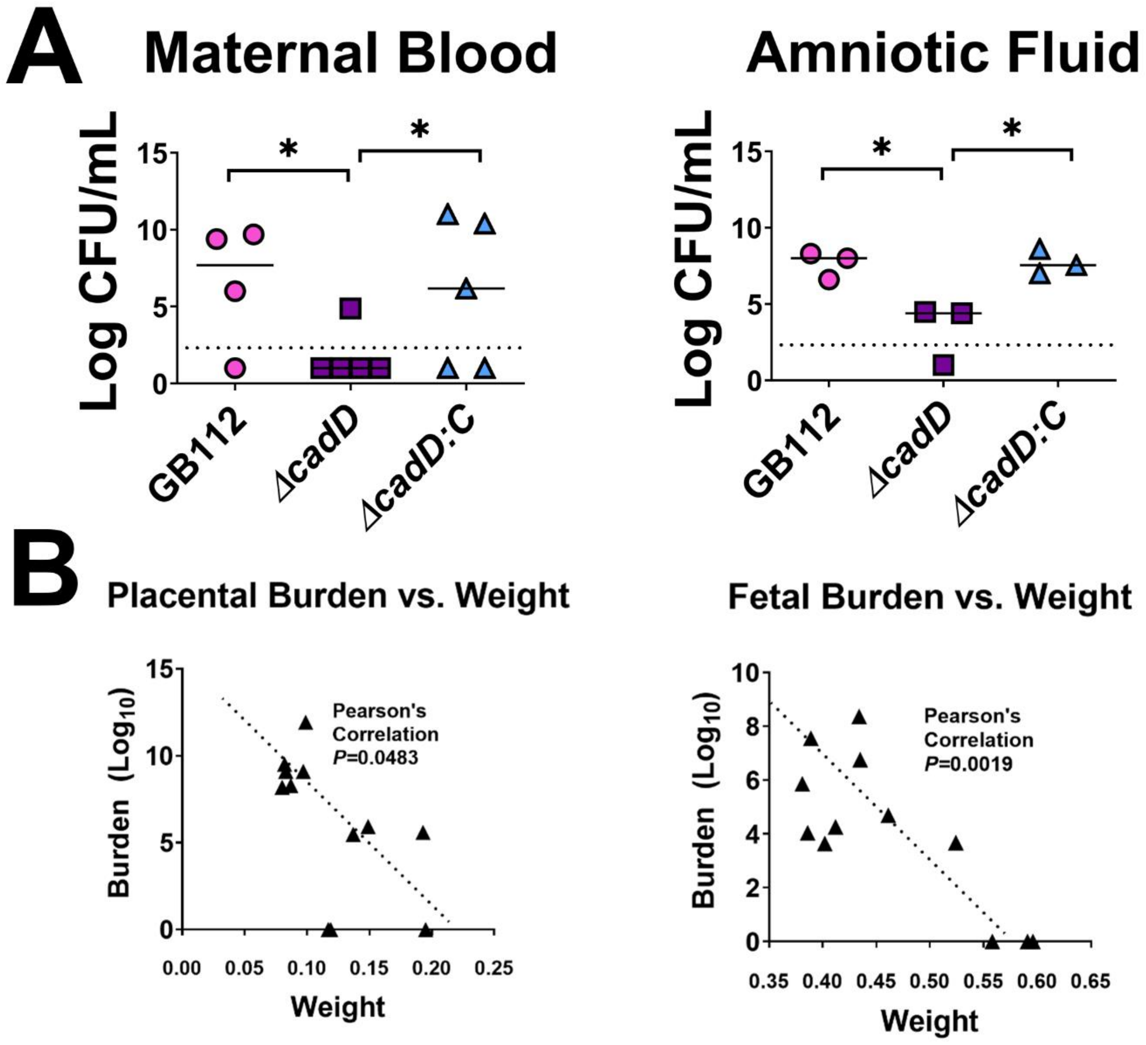

Supplemental Figure 4. The *cadD* locus is critical for GBS invasion into the amniotic cavity, maternal blood, and disruption of placental and fetal development. A) Quantitative culture (Log CFU/mL) analysis of amniotic fluid and maternal blood from pregnant mice infected with WT GB112 (pink circles), Δ*cadD* isogenic mutant (purple squares), and Δ*cadD:C* complemented mutant (blue triangles). Individual data points indicate each separate animal analyzed (either dam or fetal-placental unit). Lines indicate median. Horizontal dotted line indicates limit of detection. *P <0.05, one-way ANOVA with Tukey's *post hoc* multiple corrections test. B) High bacterial burden is inversely correlated with fetal (P=0.0019) and placental (P=0.0483) weight as determined by Pearson's correlation (n=12, individual data points indicate individual animals).

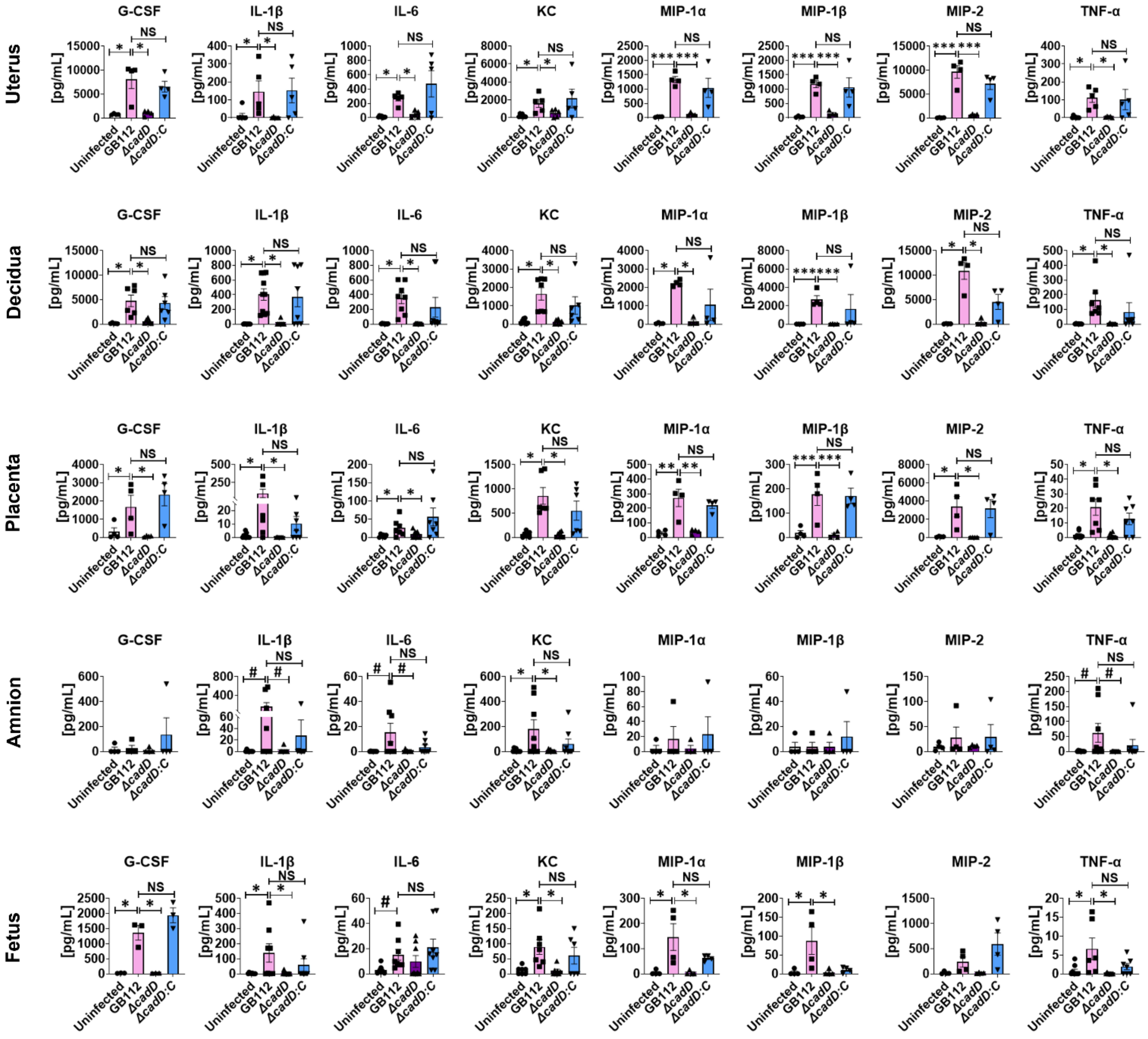

Supplemental Figure 5. Analysis of proinflammatory cytokine production in the gravid reproductive tract. Quantification of host cytokine production (G-CSF, IL-1β, IL-6, KC, MIP-1α, MIP-1β, MIP-2, TNF-α) in discrete tissue compartments (uterus, decidua, placenta, amnion, fetus) in response to GBS infection Bars represent mean cytokine concentrations +/- SEM (error bars) derived from separate dams (individual data points) for the uterus, and separate fetal-placental units (individual data points) for the decidua, placenta, amnion, and fetal tissues per group of uninfected animals (white bars), GB112-infected animals (pink bars), isogenic Δ*cadD*-infected animals (purple bars), complemented Δ*cadD:C*-infected animals (blue bars). NS= not significant,

*P<0.05, **P<0.01, ***P<0.001, ****P<0.0001, one-way ANOVA with Tukey's multiple corrections test or #P<0.05, one-tailed Student's *t* test.

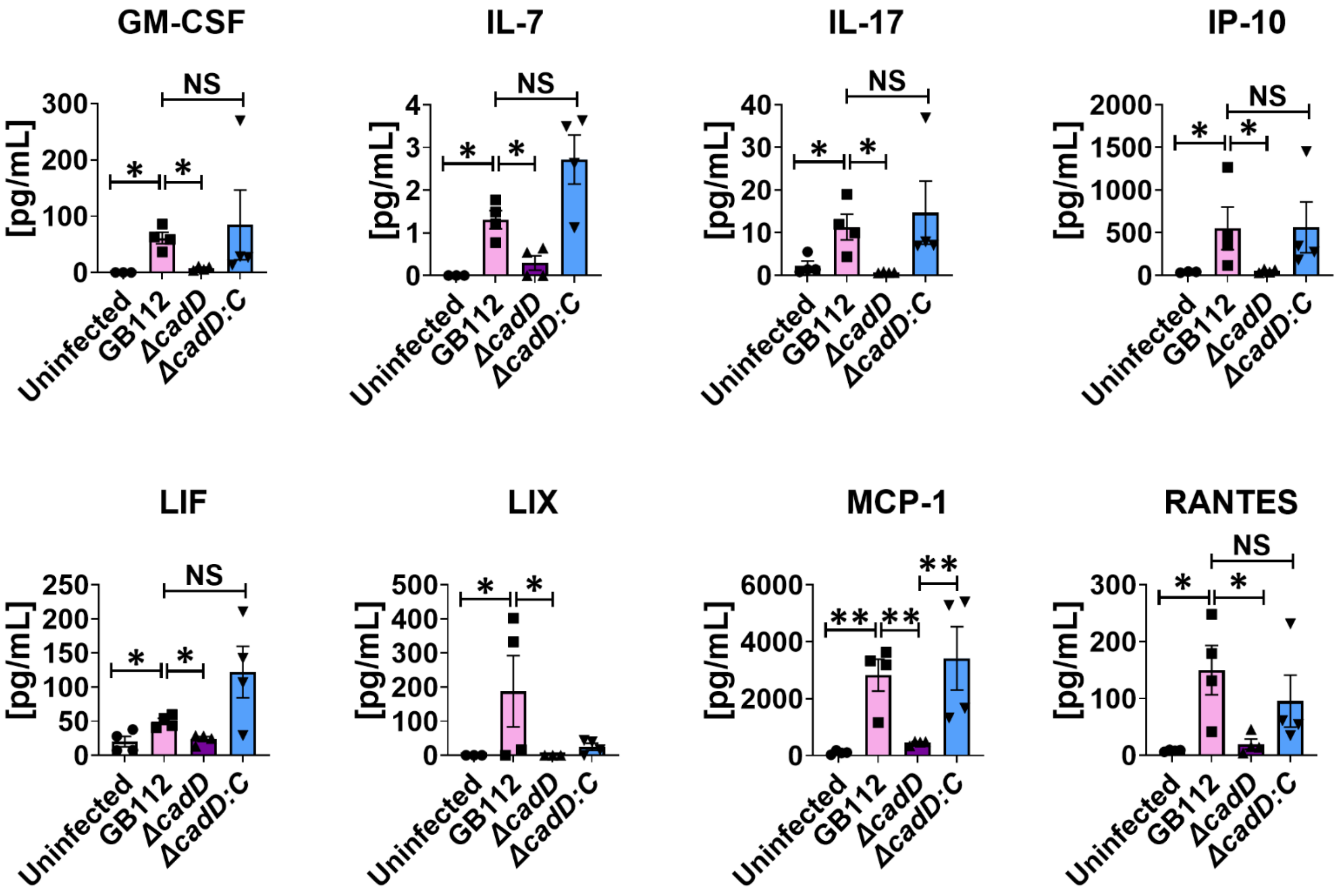

Supplemental Figure 6. Analysis of proinflammatory cytokine production in the uterus. Quantification of host cytokine production in discrete tissue compartments in response to GBS infection. Bars represent mean cytokine concentrations +/- SEM (error bars) derived from separate dams (individual data points) per group of uninfected animals uninfected animals (white bars), GB112-infected animals (pink bars), isogenic Δ*cadD*-infected animals (purple bars), complemented Δ*cadD:C*-infected animals (blue bars). NS= not significant, *P<0.05, **P<0.01, ***P<0.001, ****P<0.0001, one-way ANOVA with Tukey's multiple corrections test or #P<0.05, one-tailed Student's *t* test.

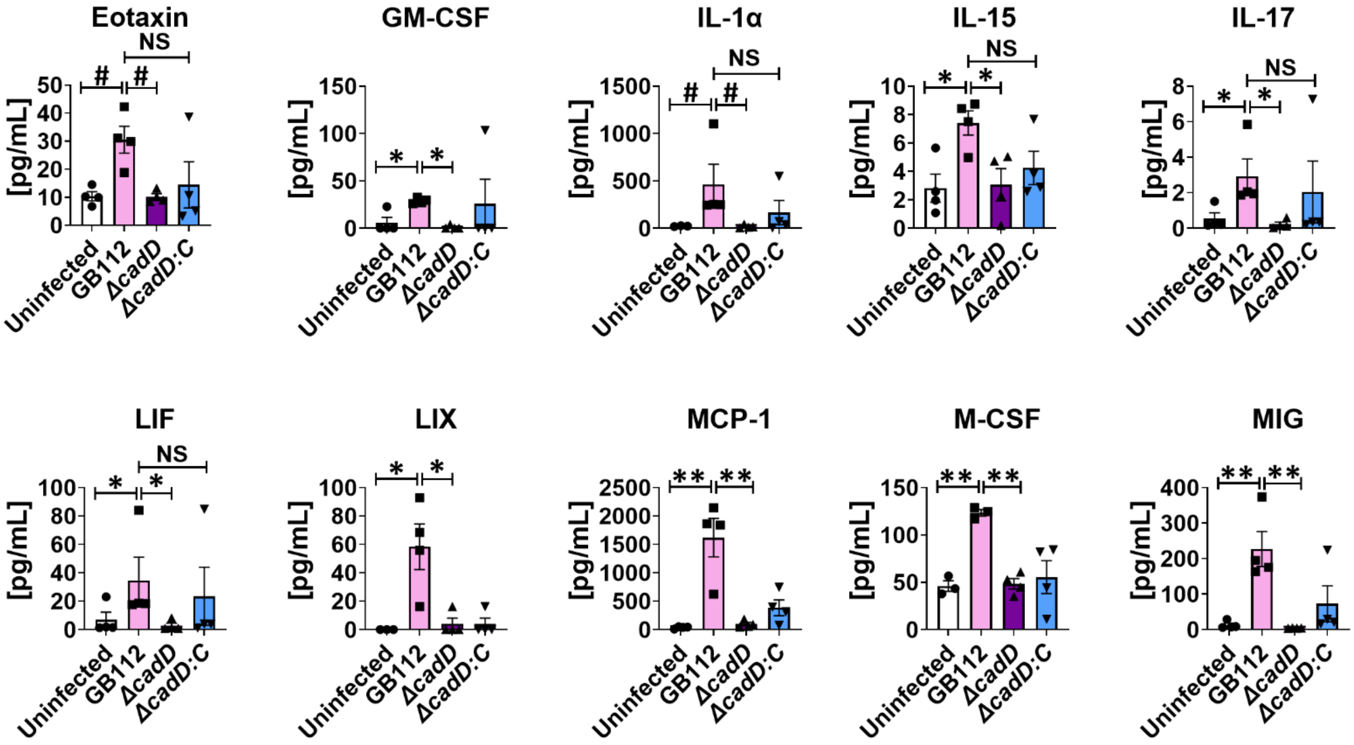

Supplemental Figure 7. Analysis of proinflammatory cytokine production in the decidua. Quantification of host cytokine production in discrete tissue compartments in response to GBS infection. Bars represent mean cytokine concentrations +/- SEM (error bars) derived from separate fetal-placental units (individual data points) per group of uninfected animals (white bars), GB112-infected animals (pink bars), isogenic Δ*cadD*-infected animals (purple bars), complemented Δ*cadD:C*-infected animals (blue bars). NS= not significant, *P<0.05, **P<0.01, ***P<0.001, ****P<0.0001, one-way ANOVA with Tukey's multiple corrections test or #P<0.05, one-tailed Student's *t* test.

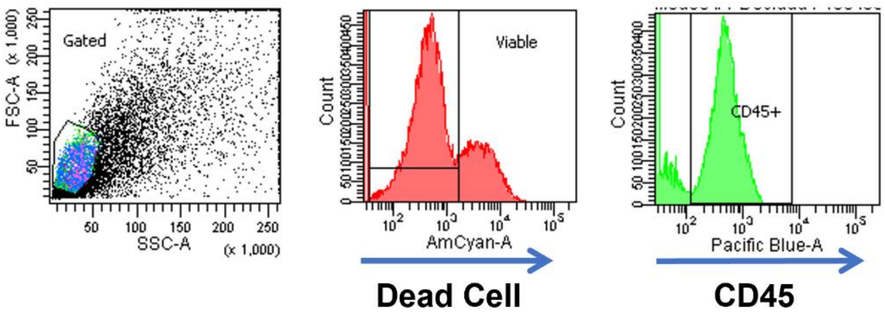

Supplemental Figure 8. Gating strategy employed for flow cytometry analyses. Total mouse placental or decidual cells were isolated by enzymatic digestion and percoll gradient centrifugation. One million cells were stained for flow cytometry. The first gate (far left panel) is a tight FSC x SSC dot plot. The second gate (middle panel) is histogram of viability dye where we gate negatively stained cells (viable) at the 10^3 on the x-axis (bimodal population). The third gate (far right panel) is a histogram of CD45 straining CD45+ cells were gated to omit non-immune cells at the 10^2 on the x-axis (bimodal population). Subsequent CD11b x F4/80 gating was utilized to identify macrophages (double positive) which falls around the 10^3-10^5 range on the x-axis for F4/80 and around 10^4 on the y-axis for CD11b.

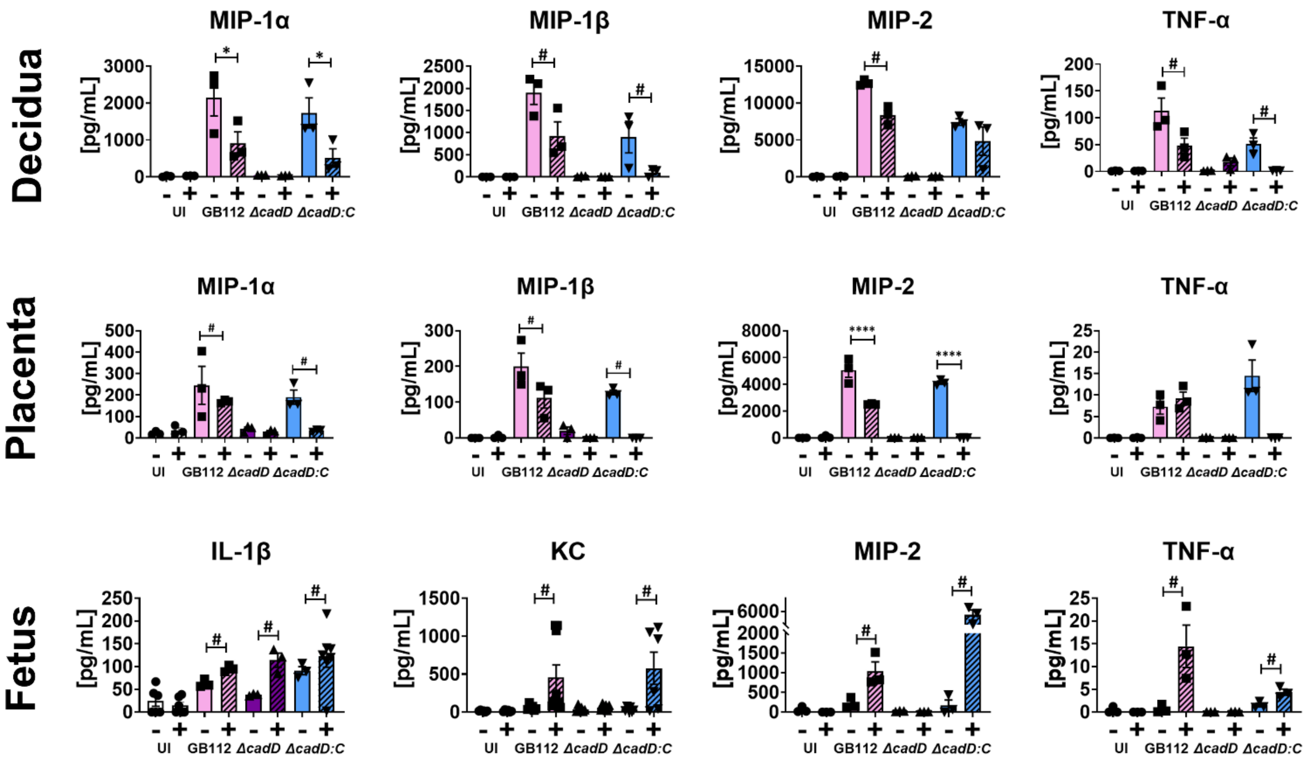

Supplemental Figure 9. Analysis of proinflammatory cytokine production in response to GBS infection with or without macrophage depletion treatments Multiplex cytokine analyses reveal differential production of cytokines (MIP-1α, MIP-1β, MIP-2 and TNF-α) in response to GBS infection and macrophage depletion. Bars represent mean cytokine concentrations +/- SEM (error bars) derived from separate fetal-placental units (individual data points) per group of uninfected animals (white bars), GB112-infected animals (pink bars), isogenic Δ*cadD*-infected animals (purple bars) and complemented Δ*cadD:C*-infected animals (blue bars). Solid bars indicate isotype control-treated animals (-), and hatched bars indicate anti-F4/80-treated animals (+). NS= not significant, *P<0.05, **P<0.01, ***P<0.001, ****P<0.0001, one-way ANOVA with Tukey's multiple corrections test or #P<0.05, one-tailed Student's *t* test.

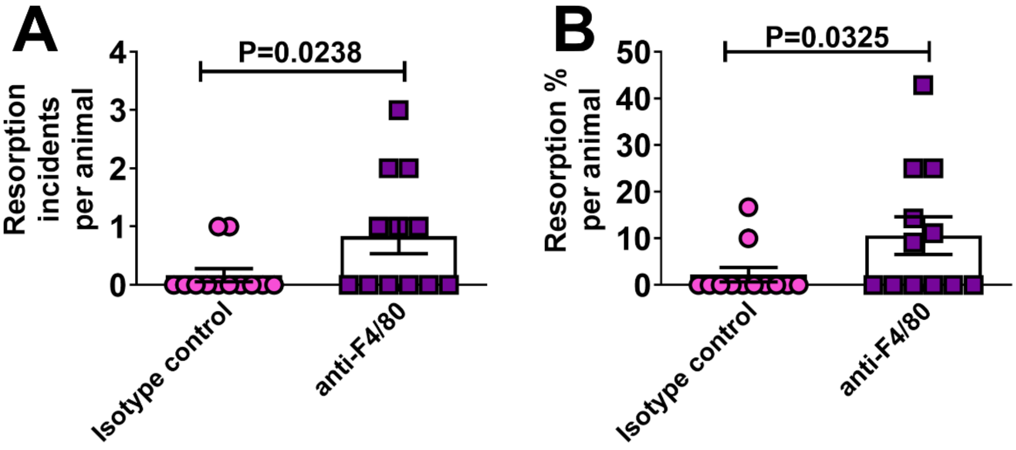

Supplemental Figure 10. Analysis of fetal resorption in response to macrophage depletion in a mouse model of GBS infection during pregnancy. Pregnant mice were treated with anti-F4/80 to deplete macrophages or an isotype antibody as a negative control. The number of fetal resorption events was calculated at embryonic day 15.5 and calculated as number of resorption events per animal (A) and as a percent with respect to total number of fetuses per gravid dam (B). Macrophage depletion by anti-F4/80 treatment (purple squares) was associated with significant increases in resorption events per animal (panel A, P=0.0238) and percent fetuses resorbed (panel B, P=0.0325) as determined by one-tailed Student's *t* test compared to isotype control animals (pink circles). Bars represent mean values +/- SEM (error bars) derived from 12 dams per experimental group.
