# Supplementary material for: Streptococcus agalactiae cadD alleviates metal stress and promotes intracellular survival in macrophages and ascending infection during pregnancy
Source: Nat Commun. 2022 Sep 14;13:5392. doi: 10.1038/s41467-022-32916-7 (PMC9474517; doi:10.1038/s41467-022-32916-7)
Supplement: Supplementary file 2 — Reporting Summary [file 41467_2022_32916_MOESM2_ESM.pdf]

nature portfolio

Corresponding author(s): Jennifer Gaddy

Last updated by author(s): 08/11/2022

Reporting Summary

Nature Portfolio wishes to improve the reproducibility of the work that we publish. This form provides structure for consistency and transparency in reporting. For further information on Nature Portfolio policies, see our Editorial Policies and the Editorial Policy Checklist.

Statistics

For all statistical analyses, confirm that the following items are present in the figure legend, table legend, main text, or Methods section.

n/a	Confirmed	
☐	☒	The exact sample size (*n*) for each experimental group/condition, given as a discrete number and unit of measurement
☐	☒	A statement on whether measurements were taken from distinct samples or whether the same sample was measured repeatedly
☐	☒	The statistical test(s) used AND whether they are one- or two-sided<br>*Only common tests should be described solely by name; describe more complex techniques in the Methods section.*
☐	☒	A description of all covariates tested
☐	☒	A description of any assumptions or corrections, such as tests of normality and adjustment for multiple comparisons
☐	☒	A full description of the statistical parameters including central tendency (e.g. means) or other basic estimates (e.g. regression coefficient) AND variation (e.g. standard deviation) or associated estimates of uncertainty (e.g. confidence intervals)
☐	☒	For null hypothesis testing, the test statistic (e.g. *F*, *t*, *r*) with confidence intervals, effect sizes, degrees of freedom and *P* value noted<br>*Give P values as exact values whenever suitable.*
☒	☐	For Bayesian analysis, information on the choice of priors and Markov chain Monte Carlo settings
☒	☐	For hierarchical and complex designs, identification of the appropriate level for tests and full reporting of outcomes
☐	☒	Estimates of effect sizes (e.g. Cohen's *d*, Pearson's *r*), indicating how they were calculated

Our web collection on statistics for biologists contains articles on many of the points above.

Software and code

Policy information about availability of computer code

Data collection	BD FACS DIVA 2 Software (BD Biosciences), ToupView software package (ToupTek Photonics), tBLASTx (NCBI/NLM), EVOS M5000 Imaging System Software (ThermoFisher),
Data analysis	GraphPad Prism 9 (GraphPad Software Inc.), MUSCLE software (Drive 5), ImageJ software (NIH), Microsoft Excel (Microsoft)

For manuscripts utilizing custom algorithms or software that are central to the research but not yet described in published literature, software must be made available to editors and reviewers. We strongly encourage code deposition in a community repository (e.g. GitHub). See the Nature Portfolio guidelines for submitting code & software for further information.

Data

Policy information about availability of data

All manuscripts must include a data availability statement. This statement should provide the following information, where applicable:
- Accession codes, unique identifiers, or web links for publicly available datasets
- A description of any restrictions on data availability
- For clinical datasets or third party data, please ensure that the statement adheres to our policy

RNA-Seq data were deposited in the Gene Expression Omnibus (accession number GSE184709).

Field-specific reporting

Please select the one below that is the best fit for your research. If you are not sure, read the appropriate sections before making your selection.

- [x] Life sciences
- [ ] Behavioural & social sciences
- [ ] Ecological, evolutionary & environmental sciences

For a reference copy of the document with all sections, see nature.com/documents/nr-reporting-summary-flat.pdf

Life sciences study design

All studies must disclose on these points even when the disclosure is negative.

Sample size	The in vitro sample size estimation was completed using a one-way ANOVA test of preliminary data. For each of the four cohorts (Uninfected vs. GB112-infected vs. ΔcadD-infected vs. ΔcadD:C) per experiment, it provides at least 80% power to detect an overall 25% difference in phenotypic changes among the study groups with two-sided type I error = 5%. Thus, we determined these experiments would need to be repeated 3 times. For all studies at least three biological replicates were utilized for each experimental procedure. Individual data points on every graph represent individual biological replicates. For animal studies, using our preliminary data, we estimated the within-group standard deviation of log-transformed CFU to be 1.5 to 2.0. We will have excellent power (87% to 98%) to detect similar treatment effects we saw in the preliminary studies between experimental cohorts. In this simple power analysis, analysis of variance model was used with family-wise type I error rate of 5%.
Data exclusions	No data were excluded from analysis.
Replication	All experiments were replicated at least three times with fresh biological replicates. All attempts at replication were successful.
Randomization	Allocation of cells/tissues/animals for experimental procedures was done at random. For histopathological and microscopical examinations samples were blinded with alpha-numeric codes to ensure unbiased imaging and analyses.
Blinding	For histopathological and microscopical examinations samples were blinded with alpha-numeric codes to ensure unbiased imaging and analyses, and for human tissue specimens all tissues were collected in a de-identified fashion. For quantitative culture, elemental analyses, growth analyses, and cytokine analyses samples were coded with alpha-numeric designations during analyses to facilitate blinded analyses.

Reporting for specific materials, systems and methods

We require information from authors about some types of materials, experimental systems and methods used in many studies. Here, indicate whether each material, system or method listed is relevant to your study. If you are not sure if a list item applies to your research, read the appropriate section before selecting a response.

Materials & experimental systems

n/a	Involved in the study	
[ ]	[x]	Antibodies
[ ]	[x]	Eukaryotic cell lines
[x]	[ ]	Palaeontology and archaeology
[ ]	[x]	Animals and other organisms
[ ]	[x]	Human research participants
[x]	[ ]	Clinical data
[x]	[ ]	Dual use research of concern

Methods

n/a	Involved in the study	
[x]	[ ]	ChIP-seq
[ ]	[x]	Flow cytometry
[x]	[ ]	MRI-based neuroimaging

Antibodies

Antibodies used	rat IgG2a kappa, clone: BM8, anti-mouse F4/80 monoclonal antibody (16-4801-86; ThermoFisher),<br>rat IgG2a kappa, clone: eBR2a, isotype control (02-9602; ThermoFisher) ,<br>rabbit polyclonal anti-GBS antibody (ab78846; Abcam)<br>rat IgG2a kappa, clone: 30-F11 anti-mouse CD45 pacific blue; (Biolegend; catalong # 103126)<br>rat IgG2b kappa, clone: M1/70 anti-mouse CD11b AF700; (eBioscience; catalog # 56-0112)<br>rat IgG2a kappa, clone BM8 anti-mouse F4/80 PE; (eBioscience; catalog #12-4801)<br>goat-anti-rabbit antibody conjugated to an Alexa Fluor 488 fluorophore (ab150077; Abcam)<br>goat-anti-rat antibody conjugated to an Alexa Fluor 594 fluorophore (ab150160; Abcam).
Validation	No primary antibody treatments were used as negative controls to ensure cross-reactivity of secondary antibodies was minimal. The polyclonal rabbit antibody to Group B Streptococcus (ab78846; Abcam) was validated by the Translational Shared Pathology Resource at Vanderbilt University Medical Center using GBS culture-positive mouse placenta and is always performed as an positive control for each run of immunohistochemical analyses. Rat IgG2a kappa, clone BM8 anti-mouse F4/80 PE; (eBioscience; catalog

#12-4801) has been validated by the manufacturer as having reactivity against mouse antigen. Rat IgG2b kappa, clone: M1/70 anti-mouse CD11b AF700; (eBioscience; catalog # 56-0112) has been validated by the manufacturer as having reactivity against mouse antigen. Rat IgG2a kappa, clone: 30-F11 anti-mouse CD45 pacific blue; (Biolegend; catalong # 103126) has been validated by the manufacturer as having reactivity against C57BL/6 mouse splenocytes. Rat IgG2a kappa, clone: BM8, anti-mouse F4/80 monoclonal antibody (16-4801-86; ThermoFisher) has been validated by the manufacturer as having reactivity against mouse antigen.

Eukaryotic cell lines

Policy information about cell lines

Cell line source(s)	THP-1 cells, ATCC
Authentication	No authentication was performed.
Mycoplasma contamination	Cell lines were not tested for Mycoplasma contamination.
Commonly misidentified lines (See ICLAC register)	No commonly misidentified lines were utilized in this study.

Animals and other organisms

Policy information about studies involving animals; ARRIVE guidelines recommended for reporting animal research

Laboratory animals	C57BL6/J mice age 6-10 weeks old, both male and female from Jackson Laboratories were utilized for harem breeding. Only pregnant female mice were used for the infection studies performed in this report.
Wild animals	No wild animals were utilized.
Field-collected samples	No field-collected samples were utilized.
Ethics oversight	All animal experiments were performed in accordance with the Animal Welfare Act, U.S. federal law, and NIH guidelines. All experiments were carried out under a protocol approved by Vanderbilt University Institutional Animal Care and Use Committee (IACUC: M/14/034 and M/17/012), a body that has been accredited by the Association of Assessment and Accreditation of Laboratory Animal Care Act (AAALAC).

Note that full information on the approval of the study protocol must also be provided in the manuscript.

Human research participants

Policy information about studies involving human research participants

Population characteristics	Placenta were collected from healthy, group B Streptococcus negative, term (38 weeks or later), non-laboring women between the ages of 18 and 40 years undergoing obstetrically indicated, scheduled, cesarean section delivery.
Recruitment	Patients were approached upon admission on the scheduled day of delivery and recruited by verbal and written informed consent. Our approved consent forms are English-language and thus, this could present a barrier for enrolling non-English speakers such as those patients from under-represented minority ethnic or racial groups.
Ethics oversight	This study was carried out in accordance with the recommendations of the Vanderbilt University Medical Center Institutional Review Board. This protocol was approved by the Institutional Review Board (IRB #181998 and #00005756).

Note that full information on the approval of the study protocol must also be provided in the manuscript.

Flow Cytometry

Plots

Confirm that:

- [x] The axis labels state the marker and fluorochrome used (e.g. CD4-FITC).
- [x] The axis scales are clearly visible. Include numbers along axes only for bottom left plot of group (a 'group' is an analysis of identical markers).
- [x] All plots are contour plots with outliers or pseudocolor plots.
- [x] A numerical value for number of cells or percentage (with statistics) is provided.

Methodology

Sample preparation	Total mouse placental or decidual cells were isolated by enzymatic digestion and percoll gradient centrifugation. One million cells were stained for flow cytometry.
Instrument	BD LSRII

Software	BD FACS Diva version 8.0.1
Cell population abundance	CD11b+F480+ placental macrophages are less than 1% of the total cells acquired; CD11b+F480+ decidual macrophages are less than 5% of the total cells acquired.
Gating strategy	1st gate is a tight FSCxSSC dot plot.<br>2nd gate is histogram of viability dye where we gate negatively stained cells (viable) at the 10^3 on the x-axis (bimodal pop).<br>3rd gate is histogram of CD45 straining where we gate CD45+ cells to omit non-immune cells at the 10^2 on the x-axis (bimodal pop).<br>4th gate is CD11b x F4/80 to find our macrophages (double positive) which falls around the 10^3-10^5 range on the x-axis for F4/80 and around 10^4 on the y-axis for CD11b.

[x] Tick this box to confirm that a figure exemplifying the gating strategy is provided in the Supplementary Information.
